# Mutational Scanning and Binding Free Energy Computations of the SARS-CoV-2 Spike Complexes with Distinct Groups of Neutralizing Antibodies: Energetic Drivers of Convergent Evolution of Binding Affinity and Immune Escape Hotspots

**DOI:** 10.3390/ijms26041507

**Published:** 2025-02-11

**Authors:** Mohammed Alshahrani, Vedant Parikh, Brandon Foley, Nishank Raisinghani, Gennady Verkhivker

**Affiliations:** 1 Keck Center for Science and Engineering, Graduate Program in Computational and Data Sciences, Schmid College of Science and Technology, Chapman University, Orange, CA 92866, USA; alshahrani@chapman.edu (M.A.); vedpar31@gmail.com (V.P.); bfoley@chapman.edu (B.F.); nishankr@stanford.edu (N.R.); 2 Department of Structural Biology, Stanford University, Stanford, CA 94305, USA; 3 Department of Biomedical and Pharmaceutical Sciences, Chapman University School of Pharmacy, Irvine, CA 92618, USA

**Keywords:** SARS-CoV-2 spike protein, Omicron variants, antibody binding, immune escape, molecular dynamics, protein stability, mutational scanning, binding energetics, evolutionary mechanisms

## Abstract

The rapid evolution of SARS-CoV-2 has led to the emergence of variants with increased immune evasion capabilities, posing significant challenges to antibody-based therapeutics and vaccines. In this study, we conducted a comprehensive structural and energetic analysis of SARS-CoV-2 spike receptor-binding domain (RBD) complexes with neutralizing antibodies from four distinct groups (A–D), including group A LY-CoV016, group B AZD8895 and REGN10933, group C LY-CoV555, and group D antibodies AZD1061, REGN10987, and LY-CoV1404. Using coarse-grained simplified simulation models, rapid energy-based mutational scanning, and rigorous MM-GBSA binding free energy calculations, we elucidated the molecular mechanisms of antibody binding and escape mechanisms, identified key binding hotspots, and explored the evolutionary strategies employed by the virus to evade neutralization. The residue-based decomposition analysis revealed energetic mechanisms and thermodynamic factors underlying the effect of mutations on antibody binding. The results demonstrate excellent qualitative agreement between the predicted binding hotspots and the latest experiments on antibody escape. These findings provide valuable insights into the molecular determinants of antibody binding and viral escape, highlighting the importance of targeting conserved epitopes and leveraging combination therapies to mitigate the risk of immune evasion.

## 1. Introduction

The SARS-CoV-2 Spike (S) glycoprotein is central to viral transmission and immune evasion, characterized by remarkable conformational flexibility [1,2,3,4,5,6,7,8,9,10,11,12,13,14,15]. Its S1 subunit includes the N-terminal domain (NTD), receptor-binding domain (RBD), and conserved subdomains SD1 and SD2. The NTD facilitates initial host cell attachment, while the RBD binds to the angiotensin-converting enzyme 2 (ACE2) receptor, transitioning between “up” and “down” conformations to modulate receptor and antibody accessibility [1,2,3,4,5,6,7,8,9,10,11,12,13,14,15]. SD1 and SD2 stabilize the prefusion state and orchestrate membrane fusion, highlighting the S protein’s adaptability and complexity [10,11,12,13,14,15,16,17,18]. Biophysical studies have revealed the thermodynamic and kinetic principles governing its functional transitions, emphasizing mechanisms that balance receptor binding, membrane fusion, and immune escape [16,17,18]. The extensive array of cryo-electron microscopy (cryo-EM) and X-ray structures of SARS-CoV-2 spike (S) protein variants of concern (VOCs) in various functional states, along with their interactions with antibodies, has provided significant insights into the virus’s adaptability [19,20,21,22,23,24,25]. These studies have demonstrated that VOCs can induce structural changes in the dynamic equilibrium of the S protein. Such alterations influence the distribution of functional states, impacting the binding affinities of the S proteins with different classes of antibodies and determining the efficacy of these antibodies in neutralizing the virus [19,20,21,22,23,24,25].

The BA.2.86 variant, a distinct sublineage of the Omicron variant, emerged in mid-2023 and rapidly drew global attention due to its remarkable genetic divergence from previously circulating strains [26,27,28,29,30]. The JN.1 variant, which evolved from the BA.2.86 lineage, represented a major step in the ongoing evolution of SARS-CoV-2. Detected in late 2023, JN.1 quickly became the dominant strain due to its increased ability to spread and evade the immune system [31,32,33]. Structural studies showed that key mutations in the RBD improved the ability to bind to the ACE2 receptor and avoid neutralizing antibodies [34,35]. KP.2, a descendant of JN.1, emerged with additional mutations, such as R346T and F456L, which further boosted its ability to evade immunity and spread more efficiently [36,37,38]. Similarly, KP.3, another subvariant of JN.1, carried mutations like Q493E and F456L, which worked together to strengthen ACE2 binding and increase resistance to antibodies [39,40,41]. These changes made KP.3 one of the fastest-spreading variants in 2024. JN.1 subvariants LB.1 (JN.1 + S:S31-, S:Q183H, S:R346T, S:F456L) and KP.2.3 (JN.1+ S:R346T, S:H146Q, S:S31-), which convergently acquired S31 deletion in addition to the above substitutions, spread as of June 2024 and contributed to immune evasion and the increased relative effective reproduction number [39,40]. These changes further enhanced immune evasion and transmissibility [39,40]. Meanwhile, the XEC variant, a recombinant strain combining elements of KS.1.1 and KP.3.3, appeared in mid-2024 with additional mutations like T22N and F59S. These mutations improved its ability to infect cells and evade immune responses, making it a potential candidate to become the next dominant strain [42,43]. The evolution of JN.1, KP.2, KP.3, and XEC variants demonstrated SARS-CoV-2’s ability to adapt through mutations that enhance its spread and immune evasion [36,37,38,39,40,41,42,43]. This ongoing evolution underscores the importance of continuous surveillance and adaptive vaccine strategies to keep pace with the virus.

The growing body of structural studies on SARS-CoV-2 antibodies has revealed critical insights into their binding competition with the ACE2 receptor [44,45,46]. These studies highlight multiple antigenic sites on the S protein, which can be targeted to achieve cross-neutralization. By synergistically targeting both conserved and variable epitopes within the receptor-binding domain (RBD), antibodies can effectively neutralize the virus, even against emerging variants [44,45,46]. SARS-CoV-2 antibodies were categorized into classes based on their binding characteristics. Class 1 and class 2 antibodies are particularly significant as they target epitopes overlapping with the ACE2 binding site, directly blocking viral attachment and entry into host cells [44,45]. Extensive research has further refined the classification of antibodies by examining their diverse binding epitopes and neutralization mechanisms [47,48,49,50,51,52,53,54,55].

High-throughput yeast display screening has been used to map RBD escape mutations for 247 human anti-RBD neutralizing antibodies, classifying them into 6 epitope groups (A–F) [56,57,58]. This classification aligns with earlier structural studies [44,45,47,53] where groups A–D correspond to RBS A–D and Class 1–2 antibodies. These antibodies target RBD residues critical for ACE2 binding, effectively blocking viral entry. Group A and B antibodies, such as LY-CoV016 and AZD8895, bind the “up” conformation, while group C and D antibodies, like LY-CoV555 and REGN-10987, bind both “up” and “down” conformations, enhancing neutralization across structural states [59]. Groups E and F, analogous to class 3 and 4 antibodies, target non-ACE2-binding epitopes, neutralizing the virus through mechanisms like stabilizing the RBD or inducing allosteric changes [45]. A recent study expanded this framework by analyzing 1640 antibodies from vaccinated individuals with BA.1 breakthrough infections, grouping them into 12 epitope categories [60]. Groups A–C target the ACE2-binding site, while group D antibodies (e.g., REGN-10987, LY-CoV1404) bind residues 440–449. Groups E and F are subdivided (E1–E3, F1–F3), with some antibodies competing with ACE2 and others neutralizing through alternative mechanisms [60]. Follow-up studies using multidimensional scaling and t-SNE mapped antibody escape profiles from BA.2, BA.5, and Omicron infections, revealing that mutations like R346T and K444N enable immune evasion in specific groups [61,62]. Another study analyzed 2688 antibodies from XBB and JN.1 infections, clustering them into 22 groups [63]. Groups A1/A2 and B directly compete with ACE2, while groups D2/D3/D4 and F3 also show strong neutralizing potential. Antibodies in groups E1/E2, E3, and F1 target non-ACE2 regions and are less effective. Notably, JN.1 reinfections induce broadly neutralizing antibodies, particularly in group F3, highlighting their importance against emerging variants [63]. Recent strategies focus on targeting non-immunodominant epitopes and conserved RBD regions to develop broadly neutralizing antibodies (Abs) [64]. Several promising antibody candidates include SA55 (group F3) and SA58 (group E1), which counter-escape mutations in variants like BQ.1, XBB, and JN.1 [64]. Antibodies in groups F3, A1, B, and D3 remain effective against JN.1 subvariants, while others (A2, D2, D4, E1/E2.1) are more prone to evasion [62,63,64]. These findings underscore the evolving antibody response to SARS-CoV-2 and the importance of targeting conserved epitopes for durable immunity. The recently discovered antibodies demonstrate exceptional neutralization against all tested SARS-CoV-2 variants, surpassing the broadly neutralizing antibody SA55 [65,66,67].

Computer simulations have become essential for understanding the SARS-CoV-2 spike (S) protein, its interactions with ACE2, and its evasion of neutralizing antibodies at the atomic level [13,68,69,70,71]. These tools provide unprecedented insights into the structural and energetic factors governing viral–host interactions and immune escape strategies. Using molecular dynamics (MD) simulations and Markov state models (MSM), the conformational landscapes of Omicron variants like XBB.1 and XBB.1.5, as well as their complexes with ACE2 and antibodies, have been systematically mapped [72]. Mutational scanning and binding analyses of XBB variants revealed epistatic interactions among key residues (e.g., Y501, R498, Q493, L455F, F456L), enhancing ACE2 binding while enabling antibody resistance [73,74]. Convergent mutations, such as F456L and F486P, further highlight the virus’s ability to balance receptor affinity and immune evasion. Integrating AlphaFold2-based predictions with ensemble analyses of S protein-ACE2 complexes for variants like JN.1, KP.1, KP.2, and KP.3 identified binding energy hotspots and epistatic interactions involving L455, F456, and Q493 [75]. These findings demonstrate how the virus maintains ACE2 binding while evading immunity.

Previous studies have also revealed that the S protein can function as an allosteric regulatory machine, using its intrinsic flexibility to regulate binding and immune evasion [76,77,78]. By combining molecular dynamics (MD) simulations, ensemble-based mutational scanning of protein stability and binding, and perturbation-based network profiling of allosteric interactions, our studies have examined the mechanisms of the S binding with antibodies [78,79] showing that antibody-escaping mutations often target structurally adaptable energy hotspots and allosteric effector centers. Recent studies have employed advanced computational approaches to explore the dynamic behavior of the S protein and its mutational landscape, providing critical insights into viral adaptation and antibody escape mechanisms [80,81]. A comprehensive analysis of the effects of the Omicron spike amino acid changes in the interaction with human antibodies was presented in an excellent study [80], and a computational workflow combining constrained logic programming and structural analysis to predict the behavior of new future mutants [81] highlight the collaborative and multidisciplinary nature of computational virology. Experimental and computational studies show that cross-neutralization against Omicron variants involves a trade-off between structural stability, binding strength, and allosteric interactions, shaping evolving escape hotspots linked to antigenic drift and convergent evolution [82,83]. These insights underscore the importance of targeting conserved regions or disrupting critical interactions for next-generation vaccines and therapeutics.

Convergent mutations at residues like R346, K444, N450, F486, and Q493 reflect selective pressures from immunity, enhancing ACE2 binding and immune evasion through electrostatic interactions [84,85,86,87]. Computational analyses of Omicron RBD-ACE2 binding emphasize the role of positively charged lysine residues (e.g., K378, R403, K440) in mediating strong electrostatic interactions with ACE2 [73,88]. Targeting conserved electrostatic hotspots or disrupting critical interactions, such as those in the S2 stem helix, has shown promise for broad neutralization [89,90].

Together, experimental and computational studies have provided compelling evidence that the cross-neutralization activity of antibodies against Omicron variants is governed by a complex and delicate balance and trade-off of multiple energetic factors and interaction contributions. These factors are linked to the evolving escape hotspots associated with antigenic drift and convergent evolution, which enable the virus to adapt to immune pressure while retaining its capacity to infect host cells. Moreover, the evolutionary trade-offs that shape the virus’s ability to balance immune evasion and ACE2 binding are complex and often antibody-dependent. While some mutations may enhance immune escape, they could also impose fitness costs that limit the virus’s transmissibility or replication efficiency. Understanding these trade-offs is critical for predicting the evolutionary trajectory of SARS-CoV-2 and designing interventions that can effectively target emerging variants. One of the key challenges lies in disentangling the contributions of individual interactions to the overall binding energy and stability of the S protein–antibody complex. Addressing these questions requires integrating high-resolution structural data with detailed dynamic and energetic analysis of the S protein binding with diverse groups and classes of antibodies. Although significant progress has been made in understanding the principles of cross-neutralization and immune evasion, the molecular and energetic details that govern these processes often lack quantifiable analysis of interactions and relative contributions of RBD residues.

In this study, we conducted a comprehensive structure-based mutational scanning of the receptor-binding domain (RBD) residues and performed binding free energy computations for the S-RBD complexes with a panel of neutralizing antibodies. These antibodies span four major classification groups—A, B, C, and D—each targeting distinct binding epitopes on the RBD. The simulated RBD–antibody complexes included: group A: CB6/LY-CoV016 (etesevimab) [91]; group B: AZD8895 [92] and REGN1033 [93]; group C: LY-CoV555 [94]; and group D: CoV2-2130/AZD1061 [92], REGN10987 [93], and LY-CoV1404 [95]. The coarse-grained (CG)-CABS (Carbon Alpha, Carbon Beta, Side Group) model [96,97,98] was used for simulations of the cryo-EM structures of the SARS-CoV-2 S complexes with the panel of antibodies. Additionally, for comparison, we performed atomistic MD simulations for the S-RBD complex with group A LY-CoV016 antibody. MD simulations are combined with mutational profiling of the RBD residues in complexes with the antibodies to enable quantitative comparison with the experimental data on antibody escape. To rigorously analyze the binding affinities of these RBD–antibody complexes, we employed the Molecular Mechanics/Generalized Born Surface Area (MM-GBSA) approach. This method allowed us to compute binding free energies and perform residue-based energy decomposition, providing detailed insights into the contributions of individual RBD residues to antibody binding. Using a combination of rapid mutational scanning with the simplified energy model and accurate binding free energy analysis using MM-GBSA, we examine the contributions of key RBD residues, with a special emphasis on convergent mutation hotspots. We also examine the balance between hydrophobic and electrostatic interactions that govern the binding of neutralizing antibodies to the S-RBD and offer a better understanding of the mechanisms governing antibody resistance.

## 2. Results and Discussion

### 2.1. Structural Analysis of S-RBD Binding with Four Classes of Antibodies A-D

**Figure 1:**
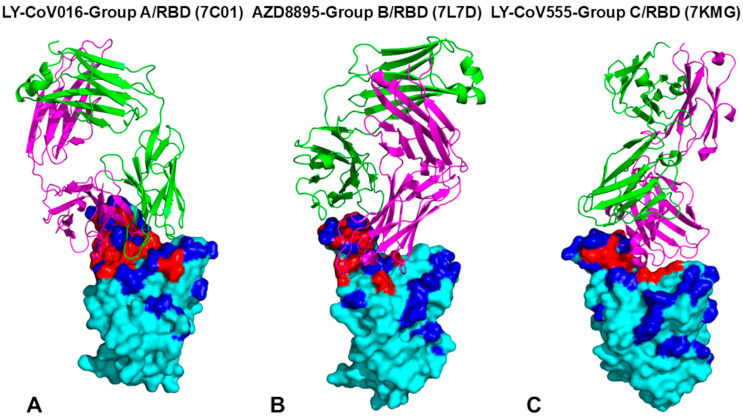
Structure and binding epitopes for the RBD complexes and binding epitopes of the A–C groups of antibodies. The overview of the group A LY-CoV016-RBD complex (**A**), group B AZD8895-RBD complex (**B**), and group C LY-CoV555-RBD complex (**C**). The RBD surface is in cyan, and the binding epitope residues are shown as the red surface. The antibody is in ribbons with a heavy chain in magenta and a light chain in green. The sites of Omicron lineages are shown as the blue surface (residues 339, 346, 356, 371, 373, 375 376, 403, 405, 408, 417, 440, 444, 445, 446, 450, 452, 455, 456, 460, 475, 477, 478, 481, 484, 486, 493, 498, 501, 505). Binding epitope residues are defined as the RBD binding interface residues that directly interact with antibodies. We used the BeAtMuSiC approach to identify binding interface residues. Residues are considered part of the interface if they are within a defined cutoff distance of 5 Å from atoms in the binding partner. Our definition of binding interface residues corresponds to the definition of the contact residues in the experimental studies.

Our study analyzed the S-RBD in complex with four groups (A–D) of neutralizing antibodies, which exhibit diverse binding mechanisms and epitope specificities impacting neutralization potency and viral escape susceptibility. Group A antibodies, like CB6/LY-CoV016, bind exclusively to the ‘up’ RBD conformation [91]. LY-CoV016’s epitope spans residues 403–505, overlapping significantly with the ACE2 binding interface, blocking viral entry via charged residues, hydrophobic contacts, and salt bridges, notably K417 (Figure 1A, Table S1) [59,91]. Escape mutations primarily affect heavy-chain CDR-interacting residues: K417, D420, L455, F456, Y473, A475, N487, G504 [59].

Group B antibodies, including AZD8895 [92] and REGN10933 [93], bind the RBD’s left shoulder (Figure 1B, Tables S2 and S3). AZD8895 interacts with residues K417–Q493, particularly F486, N487, and G476, critical for ACE2 binding but less prone to escape [59]. REGN10933 interacts with residues 403–498, forming key contacts at K417, F456, E484, F486, N487, Y489, and additional ones at Y453, L455 and Q493, influencing escape profiles [59]. Mutations at conserved sites like Y453 are minimized, while L455S and Q493E contribute to immune escape in Omicron variants JN.1, KP.2, KP.3. Overall, the evolution of escape positions tends to minimize mutations in conserved positions such as Y453 but may leverage mutations in L455 and Q493. The latter RBD sites emerged as prominent sites of mutations in the new wave of Omicron variants, including JN.1. KP.2 and KP.3 where L455S and Q493E enabled significant changes in the immune escape profile. Interestingly, two antibody cocktails—AZD8895/AZD1061 and REGN10933/REGN10987—consisting of antibodies from group B (AZD8895, REGN10933) and group D (AZD1061, REGN10987) provided complementary binding to RBD and demonstrated significant potential in preventing mutational escape. REGN10933 and REGN10987 have escaped by different mutations as mutations at F486 escaped neutralization only by REGN10933, whereas mutations at K444 escaped neutralization only by REGN10987 [99].

Group C neutralizing antibodies, such as LY-CoV555 (bamlanivimab), uniquely bind to the RBD in both ‘up’ and ‘down’ conformations, enhancing their neutralization potency [59,94]. This dual binding allows them to effectively block viral entry regardless of the RBD’s conformation. LY-CoV555’s epitope includes residues Y351–S494 (Figure 1C, Table S4), with key contacts at V483, E484, G485, and F486. These conserved hydrophobic residues are critical for RBD function and less prone to mutations. However, experimental data showed that E484 is a major escape site, with mutations like E484K (Beta variant) and E484A (Omicron sublineages) significantly reducing antibody binding and neutralization efficacy [59]. Such mutations alter the RBD’s electrostatic and structural properties, enabling immune evasion. The susceptibility to escape mutations, particularly at E484, underscores the importance of targeting conserved epitopes to counter viral evolution. The closeups of the binding epitope residues for group A LY-CoV016-RBD complex (Supplementary Materials Figure S1), group B AZD8895-RBD complex (Supplementary Materials Figure S2), and group C LY-CoV555-RBD complex (Supplementary Materials Figure S3) further illustrates similarities and differences in the interactions and composition of the binding interfaces.

**Figure 2:**
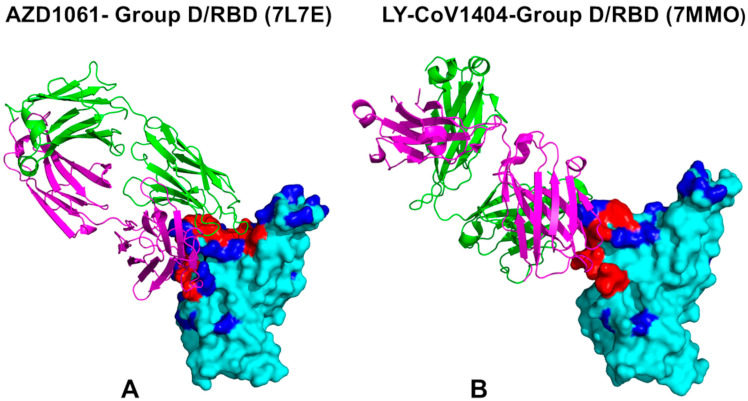
Structure and binding epitopes for the RBD complexes and binding epitopes of the group D antibodies. The overview of the AZD1061-RBD complex (**A**), and LY-CoV1404-RBD complex (**B**) The RBD surface is in cyan, and the binding epitope residues are shown as the red surface. The antibody is in ribbons with a heavy chain in magenta and a light chain in green. The sites of Omicron lineages are shown as the blue surface (residues 339, 346, 356, 371, 373, 375 376, 403, 405, 408, 417, 440, 444, 445, 446, 450, 452, 455, 456, 460, 475, 477, 478, 481, 484, 486, 493, 498, 501, 505). Binding epitope residues are defined as the RBD binding interface residues that directly interact with antibodies. We used the BeAtMuSiC contact predictor to identify binding interface residues. Residues are considered part of the interface if they are within a defined cutoff distance of 5 Å from atoms in the binding partner. Our definition of binding interface residues corresponds to the definition of the contact residues in the experimental studies.

Group D antibodies, including CoV2-2130/AZD1061 (cilgavimab) [92], REGN10987 (imdevimab) [93], and LY-CoV1404 [95], exhibit unique binding mechanisms targeting a specific loop formed by RBD residues 440–449, critical for neutralization (Figure 2). AZD1061’s epitope includes residues T345–P499 (Figure 2A, Supplementary Materials Figure S4, Table S5), with G446S impairing its neutralization against Omicron variants [59]. REGN10987 contacts RBD positions 346–501 (Table S6), forming key interactions at K444, V445, Y449, and P499. LY-CoV1404 targets an extended epitope (T345–Q506) (Figure 2B, Supplementary Materials Figure S5, Table S7), overlapping with the ACE2-binding site and engaging residues 439–450, T500, N501, and 503–506. Despite being classified as a group D binder, LY-CoV1404’s epitope resembles class 3 binder S309, with a larger contact surface area (584 Å^2^ vs. 343 Å^2^ for REGN10987) [93,95]. Electrostatic interactions involve R346 and K444, while hydrophobic contacts include L441, V445, and P499. The salt bridge between K444 and a negatively charged antibody residue is critical but disrupted by mutations like K444N, a known escape mechanism [59]. REGN10987 is susceptible to escape mutations at K444, V445, and G446. A comparison of binding epitopes and interfaces for group B REGN10933 and group D REGN10987 antibodies that bind to non-overlapping RBD regions (Supplementary Materials Figure S6) highlights important structural aspects of binding for these groups of antibodies.

### 2.2. Molecular Simulations and Collective Dynamics Reveal Role of Hinge Sites as Positions of Antibody Escape

We performed CG-CABS and all-atom MD simulations for the RBD–antibody complexes. CG-CABS trajectories were subjected to atomistic reconstruction and refinement, thereby allowing for a comparative analysis. To streamline the analysis of the RBD flexibility across different antibody complexes, we analyze and report the root-mean-square fluctuation (RMSF) profiles obtained from atomistic MD simulations (Supplementary Materials Figure S7). The RMSF profiles obtained from MD trajectories showed a highly similar conformational dynamics profile for the RBD residues (Supplementary Materials Figure S7). The simulations reproduced stability of the conserved core of the RBD antiparallel β strands (β1 to β4 and β7) (residues 354–358, 376–380, 394–403, 431–438, 507–516) and a particularly significant stabilization of β-sheets (β5 and β6) (residues 451–454 and 492–495) that anchor the RBM region to the central core (Supplementary Materials Figure S7). We found that the mobility of the RBM in the complexes with the antibodies LY-CoV016 and LY-CoV1404 is higher than with other ones, like AZD1061 and AZD8895 (Supplementary Materials Figure S7). LY-CoV016 primarily targets a broader epitope that overlaps partially with the RBM but does not fully cover or stabilize the entire RBM region. This leaves parts of the RBM relatively unconstrained, allowing for residual flexibility. In contrast, AZD8895 binds to a more focused epitope within the RBM, forming extensive interactions with residues critical for ACE2 binding. This tighter engagement reduces the mobility of the RBM. AZD1061 targets an epitope that includes both the RBM and adjacent regions, effectively clamping down on the RBM and stabilizing it further. AZD8895 and AZD1061 are larger antibodies that engage a broader surface area of the RBM and adjacent regions. Their larger size and binding orientation effectively immobilize the RBM and surrounding loops. The size and orientation of the antibody determine how much of the RBM is stabilized, with larger antibodies providing more comprehensive coverage and rigidity. The broader and less restrictive binding mode of LY-CoV016 allows for greater conformational freedom in the RBM compared to the more localized and stabilizing interactions of AZD8895 and AZD1061. The increased flexibility of the RBM in the LY-CoV016 complex also suggests that this antibody may be less effective at completely blocking ACE2 binding compared to AZD8895 and AZD1061. Residual flexibility in the RBM could allow for transient interactions with ACE2, albeit at a reduced efficiency. Conversely, the rigidification of the RBM by AZD8895 and AZD1061 ensures that the RBD is locked into a conformation that is incompatible with ACE2 binding, enhancing their neutralization potency.

We also characterized collective motions and determined the hinge regions in the SARS-CoV-2 S-RBD complexes using principal component analysis (PCA) of trajectories using the CARMA package [100]. The local minima along these profiles are typically aligned with the immobilized in global motions hinge centers, while the maxima correspond to the moving regions undergoing concerted movements leading to global changes in structure [76,79,101,102]. The low-frequency ‘soft modes’ are characterized by their cooperativity and there is a strong relationship between conformational changes and the ‘soft’ modes of motion intrinsically accessible to protein structures [76,79,101,102]. Hinge sites are regions within a protein that enable the protein to undergo conformational changes necessary for its biological functions. When hinge positions in spike–antibody complexes correspond to stable sites, several important effects can occur. Stable hinge positions may limit the flexibility of the spike protein and hinder the protein’s ability to undergo necessary conformational changes, potentially affecting its function in viral entry and immune evasion. Stability at hinge positions can influence how antibodies bind to the spike protein. If these positions are too rigid, it might reduce the effectiveness of antibody binding as the protein may not adopt the optimal conformation for interaction with the antibody. These sites are characterized by a balance between flexibility and rigidity, which is essential for maintaining structural integrity while allowing functional dynamics. In the context of the SARS-CoV-2 spike protein and its interactions with neutralizing antibodies, we will examine the pivotal role of hinge positions in coordinating the dynamics of antibody binding and viral escape.

**Figure 3:**
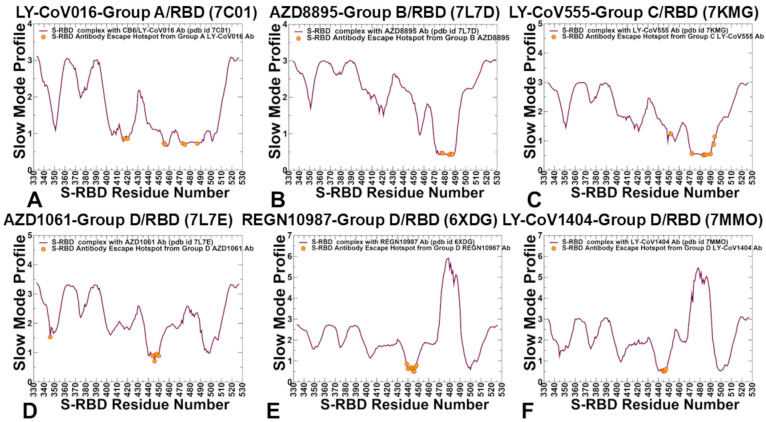
Collective dynamics of the SARS-CoV-2 S-RBD complexes with groups A–D antibodies. The mean square displacements in functional motions are averaged over the three lowest frequency modes. (**A**) The slow mode profile for the SARS-CoV-2 S-RBD complex with group A LY-CoV016 antibody (pdb id 7C01). (**B**) The slow mode profile for the SARS-CoV-2 S-RBD complex with group B AZD8895 antibody (pdb id 7L7D). (**C**) The slow mode profile for the SARS-CoV-2 S-RBD complex with group C LY-CoV555 antibody (**C**) and the slow mode profile for group D antibodies AZD1061 (**D**), REGN10987 (**E**) and LY-CoV1404 (**F**). The slow mode profiles for the SARS-CoV-2 S-RBD complexes are shown in maroon-colored lines. The antibody escape hotspots are highlighted in orange-filled circles.

PCA of MD trajectories revealed that hinge regions near the RBM (e.g., residues D405, E406, T415, K417, D420, Y421) are crucial for RBD collective motions [59]. In the presence of LY-CoV016, these regions retain flexibility, allowing small-scale RBM conformational changes due to its less restrictive binding mode. Conversely, AZD8895 and AZD1061 rigidify hinge regions, reducing RBM motions (Figure 3A). Key hinge positions include D405, E406, T415, K417, D420, Y421, L455, F456, A475, Q493, and N501, with only a few (Y421, Y453, L455, F456) corresponding to binding energy hotspots. Mutational sensitivity maps for CB6 showed minor binding energy changes for substitutions at D405, E406, K417, D420, A475, and N501, while major escape sites (K417, D420, N460, Y473, A475) align with flexible hinge sites critical for dynamics [59]. These findings underscore the role of hinge positions in coordinating antibody binding dynamics and viral escape as mutations at these sites can disrupt conformational motions, occlude epitopes, and reduce antibody effectiveness. We found that key antibody-escaping mutations can target these regulatory hinge sites, which control collective motions and allosteric interactions within the RBD. While only a small number of these sites correspond to binding energy hotspots, mutations at flexible hinge positions can significantly alter the effectiveness of antibody binding by disrupting conformational dynamics and reducing the antibody’s ability to bind effectively. Although mutations in these sites are not lethal for binding affinity, they can alter the effectiveness of the antibody as the protein may not adopt the optimal conformation for interaction with the antibody. The results showed that key antibody-escaping mutations may often target regulatory hinge positions that coordinate collective motions and allosteric interactions.

The slow mode profile for group B AZD8895-RBD revealed a broad minimum at RBD positions 473–490 (Figure 3B), with immune escape sites S477, T478, E484, G485, F486, and N487 aligning with the hinge region. For group C LY-CoV555, conserved hydrophobic residues V483, G485, F486, and E484 are critical for escape mutations, particularly E484 [59]. The slow mode profile for LY-CoV555-RBD showed local minima near residues 483–486 (Figure 3C), highlighting their role in modulating binding affinity and global antibody movements.

Structural maps of slow modes for groups A, B, and C are shown in Supplementary Materials Figure S8. Group D antibodies displayed common deep minima around K444, V445, G446, and a second minimum near T500 (Figure 3D–F). These residues, critical for immune escape [59], correspond to hinge positions modulating RBD–antibody dynamics. Structural maps for group D complexes revealed increased rigidity around residues 444–446 (Supplementary Materials Figure S9).

Of particular interest is a comparison of slow mode profiles for group D antibodies that displayed a common deep minimum around positions K444, V445, and G446 as well as a second minimum around T500 (Figure 3D–F). Although these antibodies commonly target RBD residues 440–449, which is critical for their binding and neutralization activity, group D antibodies form favorable interactions with other RBD sites. Despite the important role of 440–449 for immune escape, several sites are particularly important with many escape mutations for K444, V445, and G446 [59]. Strikingly, K444 and V445 correspond to the exact hinge positions in the complexes (Figure 3D–F), thus pointing to the important role of these residues as modulators of the RBD–antibody movements and dynamics. Structural maps of the slow mode profiles for group D antibody complexes with RBD showed increased rigidity around residues 444–446 along essential mobility modes (Supplementary Materials Figure S9). Here again, we observed that the major antibody escape center is functionally important not only for modulating binding affinity but also for global movements of the antibody around the RBD. Structural maps of the slow mode profiles illustrate the overall pattern of forming hinge sites of the RBD–antibody complexes for groups A, B, and C (Supplementary Materials Figure S8).

The binding mode of group D antibodies allows RBM mobility, modulating RBD flexibility and exposure to ACE2 interactions (Supplementary Materials Figure S9). While not targeting all ACE2-interacting residues, their epitopes overlap at K444, V445, and Y449. Mutations at K444 and V445 disrupt antibody binding and moderately affect ACE2 affinity, with Y449 being key for competitive binding due to hydrophobic interactions. AZD1061 and REGN10987 partially block ACE2 with moderate competitive potential, whereas LY-CoV1404 strongly competes by engaging T500 and N501. By forming hinge positions at 444–446 and 500–501, LY-CoV1404 effectively blocks ACE2 binding and reduces RBD dynamics, limiting its interaction with ACE2.

### 2.3. Mutational Profiling of Protein-Antibody Binding Interfaces

**Figure 4:**
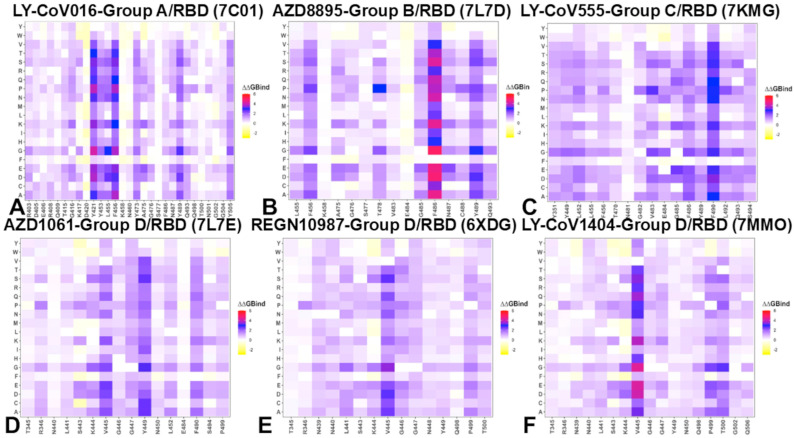
The ensemble-based mutational scanning of binding for the SARS-CoV-2 S-RBD complexes with antibodies. The mutational scanning heatmaps for the binding epitope residues in the S-RBD complexes with group A LY-CoV016 antibody (**A**), group B AZD8895 antibody (**B**), group C LY-CoV555 antibody (**C**), and group D antibodies AZD1061 (**D**), REGN10987 (**E**), and LY-CoV1404 (**F**). The binding energy hotspots correspond to residues with high mutational sensitivity. The heatmaps show the computed binding free energy changes for 20 single mutations on the sites of variants. The squares on the heatmap are colored using a 3-color scale of yellow-white-blue-red, with blue color indicating the appreciable unfavorable effects on stability and red pointing to very large destabilizing effects of mutations. The horizontal axis represents the RBD binding epitope residues. Binding epitope residues are the RBD binding interface residues that directly interact with antibodies. We used the BeAtMuSiC contact predictor to identify binding interface residues. Residues are considered part of the interface if they are within a defined cutoff distance (typically 5 Å) from atoms in the binding partner. The Y axis depicts all possible substitutions of a given RBD binding epitope residue denoting mutations to letters using a single letter annotation of the amino acid residues.

To provide a systematic comparison, we constructed mutational heatmaps for the RBD interface residues of the S complexes with the antibodies. We began by analyzing the mutational heatmap for LY-CoV016, a representative group A antibody. The heatmap revealed several strong binding hotspots where mutations cause significant destabilization of the antibody–RBD complex (Figure 4A). Y421 is surrounded by Y33, G54, G55, and S53 of the antibody’s heavy chain, forming a network of interactions critical for binding. Y453 makes a moderate number of contacts with the antibody and plays a key role in stabilizing the complex. L455 forms hydrophobic interactions with the antibody, and F456 makes a large number of contacts with M101, V98, N32, S31, S53, P100, L99, and Y33 of the heavy chain, making it a dominant binding hotspot (Figure 4A). Y489 also contributes to binding, though to a lesser extent than the other hotspots. The mutational heatmap analysis highlights the trade-off between binding affinity and immune evasion. While residues like Y421, Y453, L455, and F456 are critical for antibody binding, they are less likely to mutate due to their role in RBD stability. Instead, the virus targets more flexible residues that are less critical for ACE2 binding but sensitive to electrostatic interactions. In particular residues like K417, D420, and N460 are more flexible and sensitive to electrostatic interactions, making them prime targets for escape mutations [59].

Mutational heatmap reproduced a general trend in escaping hotspots and mutations, particularly highlighting the K417N mutation that disrupts a critical salt–bridge interaction, significantly reducing binding affinity (Figure 4A). Our analysis indicated that L455S, which alters hydrophobic contacts, can weaken antibody binding, as well as F486V, which induced considerable binding loss leading to escape. In this context, N460K combined with F486V diminishes neutralization efficacy for the BA.5 variant [59,60,61,62]. A similar pattern was seen in the mutational heatmap for group B AZD8895 where the main binding hotspots are F486, Y489, F456, and, to a lesser extent, G476 and N487 where for the latter large destabilization can occur upon N487E and N487K mutations (Figure 4B). For LY-CoV555, the largest destabilization changes are associated with mutations of F486, including F486D (ΔΔG = 5.01 kcal/mol), F4856E (ΔΔG = 4.61 kcal/mol), F486K (ΔΔG = 4.4 kcal/mol) and F486N (ΔΔG = 4.1 kcal/mol) (Figure 4B). Additionally, there are highly destabilizing mutations F456P (ΔΔG = 2.42 kcal/mol) and N487E (ΔΔG = 2.28 kcal/mol).

The experimental mutational escape data showed that group B neutralizing antibodies are very sensitive to the changes at the F486, N487, and G476 sites [59]. However, these major targeting sites of AZD8895 are critically involved in ACE2 binding and are less prone to escape. S477, T478, and E484 are found to be tolerant to mutational changes leading to moderate changes of ΔΔG < 1.0 kcal/mol, but some mutations S477N (ΔΔG = 1.19 kcal/mol), T478K (ΔΔG = 1.3 kcal/mol) and E484A (ΔΔG = 1.14 kcal/mol) are more destabilizing and are less critical for ACE2 binding. Indeed, mutations in these positions, S477N/T478K/E484A, can result in a significant escape from group B antibodies with the Omicron variant owing likely to the altered pattern of electrostatic interactions [59].

A similar mutational heatmap was obtained for another group B antibody REGN1093 (Supplementary Materials Figure S10A). The key binding hotspots are K417, Y421, Y453, L455, F456, F486, Y489, and Q493 where mutations in F456 and F486 are especially destabilizing. These results show that REGN10933 has an expanded range of strong binding hotspots compared to AZD8895, which could arguably produce a greater repertoire of escape hotspots (Figure 3B, Supplementary Materials Figure S10A). This is exactly what was found in the experimental studies [59] in which the escape profile for REGN10933 included K417, Y453, L55, Y472, T476, F486, N487, Y489, and Q493 positions. We specifically highlighted the results of mutational scanning for Omicron and particularly JN.1/KP.3 mutational sites (D339H, K356T, R403K, K417N, V445H, G446, N450D, L452W, L455S, F456L, N460K, N481K, E484K, F486P, Q493E) (Supplementary Materials Figure S10B). It can be seen that the largest destabilization changes occur upon mutations L455S, F456L, F486P, and Q493E. These results are consistent with experimental data showing that the newly emerging Omicron variants become highly resistant to group antibodies, including REGN10933 [59]. While REGN10933 has potent neutralization against earlier variants, emerging variants like JN.1 and KP.3 may exhibit reduced susceptibility to REGN10933 due to specific mutations in the RBD. Indeed, JN.1 is a descendant of the BA.2.86 lineage and carries additional mutations, including L455S, F456L, and Q493E. KP.3 is a sublineage of JN.1 and includes additional mutations such as R346T, F456L, and Q493E. Our results indicated that mutations L455S, F456L, and Q493E can disrupt antibody binding, suggesting that JN.1 and KP.3 may exhibit reduced susceptibility to REGN10933.

LY-CoV555 (bamlanivimab) is a group C neutralizing antibody, and mutational scanning of the RBD revealed that certain residues are particularly sensitive to mutations, leading to significant destabilization of the antibody–RBD complex (Figure 4C). The largest destabilizing changes were observed for F490D (ΔΔG = 3.4 kcal/mol), F490E (ΔΔG = 3.12 kcal/mol), and F490N (ΔΔG = 2.93 kcal/mol) where mutations introduce polar or charged residues, disrupting the hydrophobic interactions critical for binding. Other large destabilizing mutations included V483S (ΔΔG = 1.94 kcal/mol), which replaces a hydrophobic valine with a polar serine, weakening the binding interface as well as Q493S (ΔΔG = 1.89 kcal/mol) that disrupts hydrogen-bonding interactions, reducing binding affinity. Mutations at E484 such as E484D (ΔΔG = 1.81 kcal/mol), E484A (ΔΔG = 2.02 kcal/mol), and E484N (ΔΔG = 1.73 kcal/mol) alter the electrostatic landscape of the RBD, reducing the antibody’s binding affinity (Figure 4C). The mutational scanning results align with experimental data, showing that residues V483, E484, F486, and F490 are highly susceptible to escape mutations [59]. These positions exhibited the highest mutation escape scores, making them prime targets for viral adaptation. Although V483, F486, and F490 are conserved hydrophobic sites critical for RBD stability and ACE2 binding, the virus can still employ some mutations at these positions to evade antibodies. However, such mutations often come at a cost to viral fitness as they can destabilize the RBD or reduce ACE2 binding affinity. While residues like V483, F486, and F490 are critical for antibody binding, their role in RBD stability and ACE2 binding limits the virus’s ability to mutate these sites without compromising its infectivity. As a result, the virus tends to target second-tier binding hotspots, such as E484, which are less detrimental to ACE2 binding but still induce significant loss in antibody binding affinity. Understanding the binding escape mechanisms of LY-CoV555 provides valuable insights for therapeutic design, suggesting that antibodies engineered to target conserved hydrophobic residues like V483, F486, and F490 may be more resilient to viral evolution, as these sites are less prone to mutational changes.

For AZD1061 (cilgavimab), another group D antibody, the primary binding affinity hotspots are located at residues V445, Y449, and F490 (Figure 4D). These residues form critical interactions with the antibody, contributing to its high binding affinity and neutralization potency. Specifically, V445 and F490 are involved in hydrophobic interactions, while Y449 participates in both hydrophobic contacts and hydrogen bonding, stabilizing the antibody–RBD complex. In contrast, residues such as K444, G446, G447, and P499 play a somewhat less significant role in binding, though they still contribute to the overall interaction network. Despite their secondary role, K444 and G447 exhibit the highest experimental escape scores [59], making them dominant sites for viral escape mutations. This pattern likely arises because V445, Y449, and F490 are not only critical for antibody binding but also essential for RBD stability and ACE2 binding. As a result, mutations at these sites could compromise viral fitness, making them less prone to evolutionary changes. In contrast, residues like K444 and G447 are more flexible and less critical for RBD function, allowing the virus to exploit these positions for immune evasion without significantly affecting its ability to infect host cells.

For another group D antibody REGN10987 (imdevimab), the most significant destabilization of the antibody–RBD complex is caused by mutations at V445, particularly V445A (ΔΔG = 2.5 kcal/mol), V445D (ΔΔG = 2.65 kcal/mol), and V445E (ΔΔG = 2.26 kcal/mol) (Figure 4E, Supplementary Materials Figure S10C,D). Additionally, large destabilization changes are observed for mutations at K444, such as K444Q and K444E, as well as for mutations at P499 (Figure 4E). These residues are part of the major escape sites for REGN10987, which are dominated by K444, V445, G446, and G447 [59]. We also analyzed binding free energy changes associated with BA.2.86, JN.1, KP.2, and KP.3 mutations for REGN10987 binding (Supplementary Materials Figure S10D). The binding free energy changes showed significant loss of binding upon N440K, V445H, and G446S mutations along with somewhat smaller but still appreciable change upon L455S mutation. These key mutational changes present in JN.1, KP.2, and KP.3 variants, particularly N440K and V445H, may induce enhanced immune escape from REGN10987. Interestingly, REGN10933 and REGN10987 exhibit distinct escape pathways. Mutations at F486 specifically escape neutralization by REGN10933, while mutations at K444 escape neutralization by REGN10987. Hence, while L455S, F456L, F486P, and Q493E mutations are detrimental for REGN10933, mutations N440K, K444Q, V445H can cause escape from REGN10987. Mutations escaping both antibodies include a combination of F486V and K444Q, E484K + K444Q, and L455S + F456L: These mutations, observed in the JN.1 and KP.3 variants, can disrupt binding to both antibodies by altering the structural conformation of the RBD. The experimental evidence showed that the XBB variant that carries mutations V445P and F486S may enhance immune escape from REGN10933 and REGN10987. Similarly, our energetic analysis is consistent with the evidence that JN.1 and KP.3 variants that carry L455S, F456L, and Q493E mutations can collectively reduce the binding affinity of both antibodies.

LY-CoV1404 (bebtelovimab) is a potent group D neutralizing antibody, and mutational scanning showed that the binding energy hotspots for LY-CoV1404 are centered around residues V445, P499, T500, and K444. These residues play a crucial role in stabilizing the antibody–RBD complex through hydrophobic interactions, electrostatic contacts, and hydrogen bonding. The largest destabilizing mutations are observed for V445, including V445E (ΔΔG = 4.24 kcal/mol), V445D (ΔΔG = 4.09 kcal/mol), and V445S (ΔΔG = 3.17 kcal/mol) that disrupt the favorable hydrophobic interactions (Figure 4F). Other hydrophobic hotspots are P499 and T500 (Figure 3D). Of notice are mutations in K444 that engages in electrostatic interactions, with mutations K444D (ΔΔG = 1.93 kcal/mol) and K444E (ΔΔG = 1.64 kcal/mol) significantly reducing binding affinity (Figure 4F). The experiments showed that amino acid substitutions at K444, V445, and G446, as well as some substitutions at P499 and T500, can lead to escape from LY-CoV1404 neutralization [59]. The most important mutation, such as V445P found in the XBB variant, causes significant destabilization (ΔΔG = 3.69 kcal/mol) and is a strong escape modification K444T present in the BQ.1 variant, which leads to significant destabilization (ΔΔG = 1.78). Mutations like K444D and K444E disrupt electrostatic interactions, reducing binding affinity without compromising RBD stability or ACE2 binding. Interestingly, not all mutations at these sites lead to escape. For example, V445I (ΔΔG = 1.08 kcal/mol), V445L (ΔΔG = 0.64 kcal/mol), and G446D (ΔΔG = 0.5 kcal/mol) mutations only moderately affect antibody binding and do not result in immune escape, highlighting the nuanced nature of viral adaptation (Figure 4F). The analysis further highlights the trade-off between antibody binding and viral escape. While residues like V445, P499, and T500 are critical for antibody binding, they are also important for RBD stability and ACE2 binding, limiting the virus’s ability to mutate these sites. In contrast, charged residue K444 is less critical for ACE2 binding and prone to escape mutations due to flexibility and appreciable contribution to the antibody binding.

These findings underscore the importance of understanding the specific escape mechanisms for individual antibodies and the potential of combination therapies to enhance neutralization breadth and durability. By targeting multiple epitopes, combination therapies like REGN-COV2 can mitigate the risk of escape mutations and provide more robust protection against emerging variants. All three antibodies target V445, highlighting its importance in binding. LY-CoV1404 and REGN10987 also target K444, while AZD1061 emphasizes Y449 and F490. LY-CoV1404 and REGN10987 share similar escape pathways, with mutations at K444 and V445 being dominant, while AZD1061 is more susceptible to mutations at K444 and G447, which are less critical for RBD stability. The comparative analysis of LY-CoV1404, REGN10987, and AZD1061 reveals both shared and distinct binding and escape mechanisms. While all three antibodies target critical residues like V445 and K444, their specific binding hotspots and escape pathways differ. These differences highlight the importance of combination therapies and engineered antibodies in addressing the challenges posed by viral evolution.

### 2.4. MM-GBSA Analysis of the Binding Affinities

**Figure 5:**
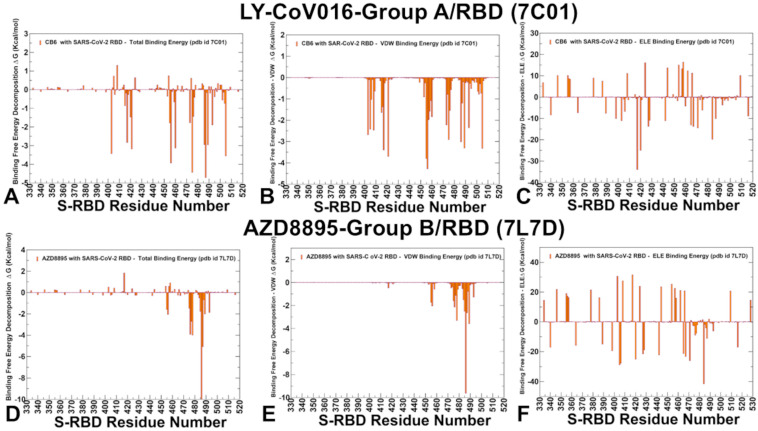
The residue-based decomposition of the binding MM-GBSA energies. The residue-based decomposition of the RBD residues for the group A LY-CoV016-RBD complex—the total binding energy (**A**), van der Waals contribution (**B**), and electrostatic interactions (**C**). The residue-based decomposition of the RBD residues for the group B AZD8895-RBD complex—the total binding energy (**D**), van der Waals contribution (**E**), and electrostatic interactions (**F**). The MM-GBSA contributions are evaluated using 1000 samples from atomistic simulations of the respective RBD-ACE2 complexes.

We utilized conformational ensembles derived from MD simulations to compute the binding free energies of RBD–antibody complexes using the MM-GBSA (Molecular Mechanics/Generalized Born Surface Area) method. This approach allowed us to perform a detailed binding free energy decomposition, examining the energetic contributions of individual RBD epitope residues. Through this analysis, we identified the binding hotspots that are critical for antibody binding and quantified the roles of van der Waals interactions and electrostatic interactions in the binding mechanism. In the MM-GBSA calculations, we investigated whether the binding affinities and contributions of the major binding hotspots are primarily determined by van der Waals interactions or electrostatic interactions. Additionally, we explored whether positions of immune escape could be associated with binding hotspots where different energetic contributions act synergistically, leading to significant loss of binding upon mutations. Through residue-based binding free energy decomposition, we identified the key residues contributing to the antibody’s binding affinity and explored the mechanisms underlying susceptibility to escape mutations. For group A LY-CoV016 antibody, the residue decomposition analysis revealed that the most favorable total binding energies are associated with the following residues: N487 (ΔG = −4.71 kcal/mol), A475 (ΔG = −4.43 kcal/mol), F456 (ΔG = −3.93 kcal/mol), R403 (ΔG = −3.42 kcal/mol), N460 (ΔG = −3.13 kcal/mol), and K417 (ΔG = −2.83 kcal/mol) (Figure 5A). The most favorable van der Waals interactions are formed by F456, L455, Y421, Y489, and K417. These residues participate in hydrophobic contacts, which are essential for stabilizing the antibody–RBD complex (Figure 5B). The largest electrostatic contributions come from K417, followed by D420, E484, and R403. These residues form charged interactions and hydrogen bonds with the antibody, enhancing binding affinity (Figure 5C). Interestingly, K417 exhibits both favorable van der Waals and electrostatic interactions, which can act synergistically to contribute to the antibody’s binding affinity. While the total binding energies are more favorable for residues like N487 and F456, the electrostatic interactions at K417 play a critical role in determining the antibody’s susceptibility to escape mutations.

The experimental escape scores are consistent with our computational findings, identifying K417, N460, A475, D420, F456, and N487 as the dominant escape hotspots [59]. These residues are frequently mutated in emerging variants, enabling the virus to evade neutralization. The binding and escape mechanisms of LY-CoV016 highlight the importance of electrostatic interactions and hydrophobic contacts in determining antibody binding affinity and susceptibility to escape mutations. While residues like N487 and F456 contribute significantly to binding, K417 plays a dual role, with both van der Waals and electrostatic interactions driving its importance as an escape hotspot.

For AZD8895, a representative group B antibody, the largest total binding energies for AZD8895 are associated with F486 (ΔG = −10.0 kcal/mol), N487 (ΔG = −5.07 kcal/mol), T478 (ΔG = −3.99 kcal/mol), G476 (ΔG = −3.93 kcal/mol), S477 (ΔG = −2.7 kcal/mol and F456 (ΔG = −2.04 kcal/mol) (Figure 5D). The most favorable van der Waals interactions are formed by F486, Y489, F456, T478, and N487. These residues participate in hydrophobic contacts, which are essential for stabilizing the antibody–RBD complex (Figure 5E). The largest electrostatic contributions come from E484, D405, E406, D429, and D442 (Figure 5F). However, the favorable electrostatic interactions at these sites are largely offset by desolvation penalties, resulting in moderate total binding energies. The results showed that F486 and N487 have the strongest binding energies but are less prone to mutations due to their critical role in RBD function and ACE2 binding. At the same time, second-tier binding hotspots T478 (ΔG = −3.99 kcal/mol) and S477 (ΔG = −2.7 kcal/mol) have favorable binding energies due to both van der Waals and electrostatic interactions (Figure 5D–F). While their contributions are weaker than those of F486 and N487, they are more flexible and less critical for RBD stability, making them prime targets for escape mutations.

Our results suggest that the virus can exploit multiple second-tier binding hotspots, such as T478 and S477, to evolve mutations that collectively reduce antibody binding. This strategy allows the virus to evade neutralization without compromising its ability to bind ACE2 [59]. The binding and escape mechanisms of AZD8895 highlight the importance of hydrophobic interactions and electrostatic contributions in determining antibody binding affinity and susceptibility to escape mutations. While residues like F486 and N487 are critical for binding, their role in RBD stability and ACE2 binding limits the virus’s ability to mutate these sites. Instead, the virus targets second-tier binding hotspots, such as T478 and S477, to evolve escape mutations that collectively reduce antibody binding.

**Figure 6:**
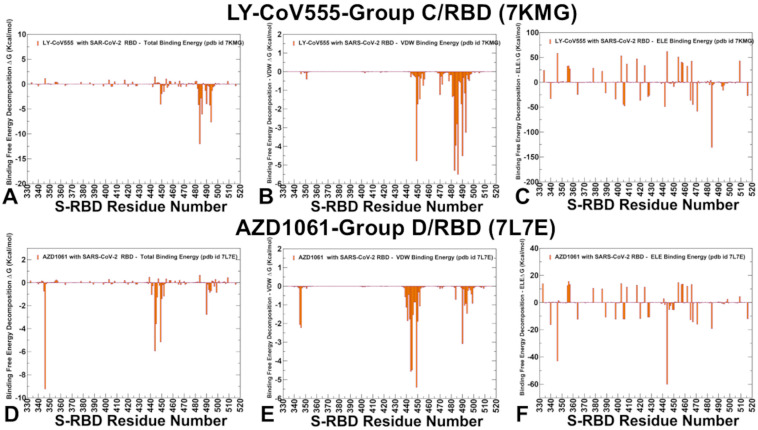
The residue-based decomposition of the binding MM-GBSA energies. The residue-based decomposition of the RBD residues for the group C LY-CoV555-RBD complex—the total binding energy (**A**), van der Waals contribution (**B**), and electrostatic interactions (**C**). The residue-based decomposition of the RBD residues for the group D AZD1061-RBD complex—the total binding energy (**D**), van der Waals contribution (**E**), and electrostatic interactions (**F**). The MM-GBSA contributions are evaluated using 1000 samples from MD simulations of the respective RBD-ACE2 complexes.

The binding energy analysis revealed several critical hotspots for group C antibody LY-CoV555, including a cluster of nearby residues: E484, S494, F486, Q493, V483, F490, and G485, as well as a standalonepeak for Y449 (Figure 6A). E484 position corresponds to the deepest and most significant binding energy peak, contributing ΔG = −12.03 kcal/mol. E484 engages in both electrostatic interactions (ΔG = −3.95 kcal/mol) and van der Waals interactions, making it a dominant binding hotspot. S494 residue contributes significantly to binding, with ΔG = −7.64 kcal/mol, though less than E484. The residue decomposition showed that F486, V483, Y449, G485, and F490 contribute favorably through van der Waals interactions, stabilizing the antibody–RBD complex (Figure 6B), while the strongest electrostatic interactions are observed at E484, followed by D442, E406, D405, and D420 (Figure 6C). The binding energy hotspots identified in our computational analysis are in line with experimental escape profiles. Indeed, E484 is the dominant escape hotspot, with mutations like E484K and E484A significantly reducing binding affinity and neutralization efficacy. V483, F486, F490, Q493, and S494 also serve as escape positions, though to a lesser extent than E484 (Figure 6A–C).

**Figure 7:**
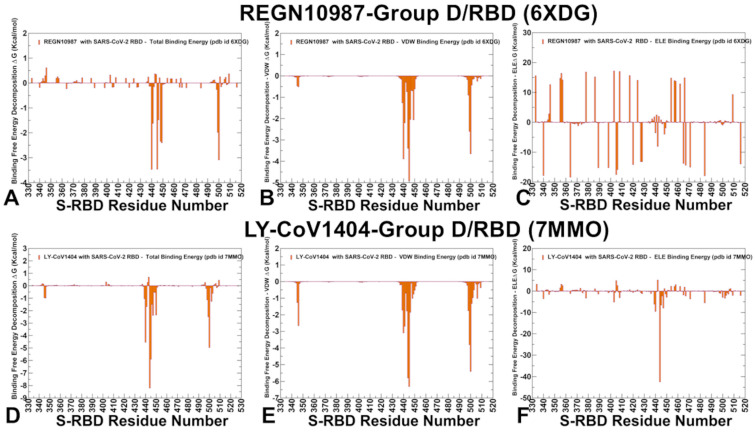
The residue-based decomposition of the binding MM-GBSA energies. The residue-based decomposition of the RBD residues for the group D REGN10987 -RBD complex—the total binding energy (**A**), van der Waals contribution (**B**), and electrostatic interactions (**C**). The residue-based decomposition of the RBD residues for the group D LY-C oV1404-RBD complex—the total binding energy (**D**), van der Waals contribution (**E**), and electrostatic interactions (**F**). The MM-GBSA contributions are evaluated using 1000 samples from MD simulations of the respective RBD-ACE2 complexes.

These residues are predicted as secondary binding hotspots, contributing to the antibody’s overall binding affinity. The results indicate that the predicted binding hotspots are strong indicators of potential escape centers for LY-CoV555. This is particularly evident for E484, which not only contributes the most favorable binding energy (Figure 6A–C) but also exhibits the highest escape mutation scores. While residues like E484 dominate the binding landscape, secondary hotspots such as V483, F486, F490, Q493, and S494 also play critical roles in both binding and immune evasion (Figure 6A–C). The virus exploits these hotspots to evolve mutations that reduce antibody binding while maintaining its ability to infect host cells. MM-GBSA computations for group D antibodies AZD1061 (cilgavimab) (Figure 6D–F), REGN10987 (imdevimab) (Figure 7A–C), and LY-CoV1404 (bebtelovimab) (Figure 7D–F) provided interesting information regarding common and unique binding hotspots and corresponding escape profiles. For AZD1061 (Cilgavimab) the key energy hotspots are R346 (ΔG = −9.23 kcal/mol), which is the most pronounced binding hotspot, contributing significantly to binding affinity (Figure 6D). Other hotspots of binding are K444 (ΔG = −5.94 kcal/mol) with strong van der Waals (ΔG = −4.55 kcal/mol) (Figure 6E) and electrostatic interactions (Figure 6F). Y449 (ΔG = −5.15 kcal/mol) and V445 (ΔG = −3.57 kcal/mol) contribute mostly through hydrophobic interactions (Figure 6E).

For REGN10987 (Imdevimab) the identified hotspots are N440, V445, T500, Y449, N448, P499, L441, and G446 (Figure 7A), with the strongest Van der Waals interactions for V445, N440, T500, P499, L441, and Y449 (Figure 7B). The electrostatic Interactions favor D364, E484, E340, D405, E406, D398, E471, and D40 where the residue distribution showed many RBD residues with favorable contributions, while many other RBD sites displayed highly unfavorable electrostatic contacts (Figure 7C). Overall, the electrostatic component is quite noisy for REGN10987 (Figure 7C) and most of the binding hotspots (N440, V445, N448) revealed complex balance and synergistic effects of hydrophobic and electrostatic contributions. For LY-CoV1404 (Bebtelovimab) K444 (ΔG = −8.2 kcal/mol) and V445 (ΔG = −5.9 kcal/mol) are the strongest binding hotspots (Figure 7D), with significant contributions from both van der Waals and electrostatic interactions (Figure 7E,F). T500, N440, P499, G447, N450, and L441 also contribute to binding, though to a lesser extent. A comparative analysis of energy contributions for group D antibodies showed that AZD1061 and LY-CoV1404 rely heavily on K444 and V445 for binding, with strong contributions from both van der Waals and electrostatic interactions (Figure 6D–F and Figure 7D–F), whereas REGN10987 exhibits a more balanced binding profile, with significant contributions from multiple residues, including N440, K444, V445, and P499 (Figure 7A–C). These results agree with and explain the experimental escape pathways where K444 is a dominant escape hotspot for all three antibodies, with mutations like K444Q and K444T significantly reducing binding affinity. V445 and G446 are also critical escape sites, particularly for LY-CoV1404 and AZD1061. LY-CoV1404 binds to a highly conserved region of the RBD with the antibody forming interactions with key residues, K444 and V445, which are critical for binding but less likely to mutate due to their role in RBD stability and ACE2 binding. As a result, LY-CoV1404 is less susceptible to escape mutations that have nullified the activity of other antibodies. For example, mutations like K444Q and V445A only moderately affect binding, whereas they significantly reduce the efficacy of other antibodies.

To summarize, the MM-GBSA binding free energy analysis, combined with residue-based decomposition, provides critical insights into the binding mechanisms and escape pathways of SARS-CoV-2 RBD–antibody complexes across groups A-D. Both van der Waals and electrostatic interactions play essential roles in stabilizing these complexes, though their contributions vary by antibody group and specific residues. Hydrophobic contacts, mediated by residues such as F456, F486, and V445, are crucial for binding, particularly in antibodies like LY-CoV016 (group A) and AZD8895 (group B). Electrostatic interactions, mediated by hotspots like K417, E484, and K444, contribute significantly through hydrogen bonds and salt bridges. For example, K417 in LY-CoV016 exhibits both van der Waals and electrostatic interactions, highlighting its dual role in binding and immune evasion. Similarly, E484 in LY-CoV555 (group C) and K444 in AZD1061 and LY-CoV1404 (group D) are critical electrostatic hotspots. Mutations at these sites disrupt both interaction types, reducing binding affinity and enabling immune escape. These findings align with experimental data identifying residues like K417, E484, and K444 as dominant escape hotspots. Recent studies on antibodies SA55 and S58 validate our computational results, showing that mutations at R346, K444, L455, and F456 significantly reduce binding affinity. For instance, the F486P mutation in Omicron subvariants drastically diminishes antibody binding, consistent with our predictions. Experimental studies further confirm that mutations at R346 and K444 lead to substantial immune evasion, corroborating our identification of these residues as key determinants of viral escape. Residue-based decomposition revealed how mutations disrupt interactions. Group B antibodies rely on F486, N487, and G476, while group D antibodies escape via mutations at K444 and V445. These insights underscore the importance of targeting conserved epitopes and using combination therapies to mitigate immune evasion. Leveraging antibodies with distinct epitope specificities reduces the likelihood of viral escape.

While this study focuses on several representative antibodies from different groups, the insights gained from our analysis have broader implications for understanding immune evasion and guiding therapeutic development. Future studies should expand the scope to include a broader range of antibodies and variants, particularly those targeting non-overlapping epitopes or exhibiting broad neutralization capabilities. Additionally, the integration of high-throughput experimental assays with computational predictions will be essential to validate findings across diverse systems and ensure their translational relevance. By leveraging the computational framework developed in this study, we can rapidly assess the immune escape potential of newly emerging variants and inform the design of next-generation vaccines and antibody therapies.

## 3. Materials and Methods

### 3.1. Structure Analysis

The crystal and cryo-EM structures of the RBD–antibody complexes for groups A–D are obtained from the Protein Data Bank [103]. The unequal distribution of complexes across the four classes reflects a combination of factors related to data availability, structural uniqueness, and functional relevance: The number of available high-quality structures in the PDB varies significantly across antibody classes. Group A and group C antibodies are represented by fewer distinct structural templates because their binding mechanisms and epitopes are relatively conserved. In contrast, Groups B and D exhibit greater diversity in their binding modes and epitope specificities, necessitating the inclusion of additional structures to capture this variability. Our primary goal was to select complexes that best represent the unique characteristics of each class while avoiding redundancy. For group A, the CB6/LY-CoV016 structure was chosen because it is widely regarded as a prototypical example of ‘up’-conformation-specific binding. This classification is supported by structural studies demonstrating its exclusive interaction with the RBD in the ‘up’ conformation, which is critical for ACE2 receptor engagement [93]. Similarly, LY-CoV555 (group C) was selected as a representative dual-conformation binder due to its well-documented ability to recognize both ‘up’ and ‘down’ conformations of the RBD, a mechanism that distinguishes it from other antibodies in this class [94]. In contrast, groups B and D exhibit more nuanced differences in their binding orientations and epitope coverage. These considerations guided our selection of representative complexes for each group. It is important to acknowledge that some of the cryo-EM and X-ray c structures analyzed in this study featured moderate-to-high resolution (ranging from 2.8 Å to 3.5 Å). While these resolutions are sufficient to identify key interactions and overall binding orientations, finer details such as water-mediated hydrogen bonds or subtle side-chain rearrangements may not be fully resolved. To account for these limitations, we relied on consensus patterns observed across multiple structures within each group and cross-referenced our findings with biochemical and mutagenesis data where available. This approach ensures that our interpretations remain robust despite the inherent resolution constraints.

Hydrogen atoms and missing residues were initially added and assigned according to the WHATIF program web interface [104]. The structures were further pre-processed through the Protein Preparation Wizard (Schrödinger, LLC, New York, NY, USA) and included the check of bond order, assignment and adjustment of ionization states, formation of disulphide bonds, removal of crystallographic water molecules and co-factors, capping of the termini, assignment of partial charges, and addition of possible missing atoms and side chains that were not assigned in the initial processing with the WHATIF program [104]. The missing loops in the cryo-EM structures were also reconstructed using template-based loop prediction approach ArchPRED [105]. The side chain rotamers were refined and optimized by the SCWRL4 tool [106]. The protonation states for all the titratable residues of the proteins were predicted at pH 7.0 using Propka 3.1 software and web server [107,108]. While the physiological pH is typically 7.4, the choice of pH 7.0 was made to ensure consistency with previous computational studies and the validated accuracy of pKa prediction tools at this pH. To assess the robustness of our results, we performed a sensitivity analysis by repeating the protonation state assignments and MM-GBSA binding free energy calculations at pH 7.4. The protein structures were then optimized using atomic-level energy minimization with composite physics and knowledge-based force fields implemented in the 3Drefine method [109,110].

### 3.2. Molecular Simulations

CG models are computationally effective approaches for simulations of large systems over long timescales. In this study, the CG-CABS model [96,97,98] was used for simulations of the SARS-CoV-2 S complexes with antibodies. In this model, the amino acid residues are represented by Cα, Cβ, the center of mass of side chains, and another pseudo atom placed in the center of the Cα-Cα pseudo-bond [96,97,98]. The position of Cα atoms is confined to a cubic lattice of a grid equal to 0.61 Å. The position of the side chain is dependent on the Cα−Cα−Cα angle of the main chain and the amino acid type. We used the CABS model as this is a high-resolution knowledge-based CG force field that is based on potentials of the mean force obtained from statistical analysis of known protein structures and structural correlations of solved protein structures [96,97,98]. The sampling scheme of the CABS model used in our study is based on Monte Carlo replica-exchange dynamics and is modeled as a long random sequence of small local moves of individual amino acids in the protein structure as well as moves of small fragments consisting of two and three residues. The default settings were used for CG-CABS simulations in which soft native-like restraints are imposed only on pairs of residues with the distance between their *C*_α_ atoms smaller than 8 Å and both residues being part of the same secondary structure elements. No additional custom-designed distance restraints were applied to the simulation scheme. CABS-flex standalone package dynamics, implemented as a Python 2.7 object-oriented package, were used for fast simulations of protein structures [98]. A series of independent CG-CABS replica-exchange simulations were performed for each of the systems studied. In each simulation, the total number of cycles was set to 10,000 and the number of cycles between trajectory frames was 100. The conformational ensembles were subjected to MODELLER-based all-atom reconstruction, including hydrogen atoms to produce atomistic models of simulation trajectories [111,112].

### 3.3. All-Atom Molecular Dynamics Simulations

All-atom MD simulations were performed for the RBD–antibody complexes. NAMD 2.13-multicore-CUDA package [113] with CHARMM36 force field [114] was employed to perform 1 µs all-atom MD simulations for the RBD–antibody complexes. The structures of the complexes were prepared in Visual Molecular Dynamics (VMD 1.9.3) [115] and with the CHARMM-GUI web server [116,117] using the Solutions Builder tool. Hydrogen atoms were modeled onto the structures prior to solvation with TIP3P water molecules [118] in a periodic box that extended 10 Å beyond any protein atom in the system. To neutralize the biological system before the simulation, Na^+^ and Cl^−^ ions were added in physiological concentrations to achieve charge neutrality, and a salt concentration of 150 mM of NaCl was used to mimic physiological concentration. All Na^+^ and Cl^−^ ions were placed at least 8 Å away from any protein atoms and from each other. MD simulations are typically performed in an aqueous environment in which the number of ions remains fixed for the duration of the simulation, with a minimally neutralizing ion environment or salt pairs to match the macroscopic salt concentration [119].

First, minimization was performed for 100,000 steps with all the hydrogen-containing bonds constrained and the protein atoms fixed. In the second stage, minimization was performed for 50,000 steps with all the protein backbone atoms fixed and for an additional 10,000 steps with no fixed atoms. The equilibration was performed for 1 ns by gradually increasing the system temperature in steps of 20 K, increasing from 10 K to 310 K, and at each step maintaining a restraint of 10 kcal mol^−1^ Å^−2^ on the protein C_α_ atoms. After the restraints on the protein atoms were removed, the system was equilibrated for an additional 10 ns. Long-range, non-bonded van der Waals interactions were computed using an atom-based cutoff of 12 Å, with the switching function beginning at 10 Å and reaching zero at 14 Å. The SHAKE method was used to constrain all the bonds associated with hydrogen atoms. The simulations were run using a leap-frog integrator with a 2 fs integration time step. The ShakeH algorithm in NAMD was applied for the water molecule constraints. The long-range electrostatic interactions were calculated using the particle mesh Ewald method [120] with a cutoff of 1.0 nm and a fourth-order (cubic) interpolation. The simulations were performed under an NPT ensemble with a Langevin thermostat and a Nosé–Hoover Langevin piston at 310 K and 1 atm. The damping coefficient (gamma) of the Langevin thermostat was 1/ps. In NAMD, the Nosé–Hoover Langevin piston method is a combination of the Nosé–Hoover constant pressure method [121] and piston fluctuation control implemented using Langevin dynamics [122,123]. An NPT production simulation was run on equilibrated structures for 1µs, keeping the temperature at 310 K and a constant pressure (1 atm).

### 3.4. Binding Free Energy Computations: Mutational Scanning and Sensitivity Analysis

We conducted mutational scanning analysis of the binding epitope residues for the S RBD–antibody complexes. Each binding epitope residue was systematically mutated using all substitutions and corresponding protein stability and binding free energy changes were computed. BeAtMuSiC approach [124,125] was employed based on statistical potentials describing the pairwise inter-residue distances, backbone torsion angles, and solvent accessibilities, considering the effect of the mutation on the strength of the interactions at the interface and on the overall stability of the complex. The binding free energy of a protein–protein complex can be expressed as the difference in the folding free energy of the complex and folding free energies of the two protein binding partners:

(1) $$\Delta G_{bind}=G^{com}-G^{A}-G^{B}$$

The change of the binding energy due to a mutation was calculated as the following:

(2) $$\Delta \Delta G_{bind}=\Delta G_{bind}^{mut}-\Delta G_{bind}^{wt}$$

We leveraged rapid calculations based on statistical potentials to compute the ensemble-averaged binding free energy changes using equilibrium samples from simulation trajectories. The binding free energy changes were obtained by averaging the results over 1000 and 10,000 equilibrium samples for each of the systems studied.

### 3.5. Binding Free Energy Computations

We calculated the ensemble-averaged changes in binding free energy using 1000 equilibrium samples obtained from simulation trajectories for each system under study. Initially, the binding free energies of the RBD–antibody complexes were assessed using the MM-GBSA approach [126,127]. Additionally, we conducted an energy decomposition analysis to evaluate the contribution of each amino acid during the binding of RBD to antibodies [128,129].

The binding free energy for the RBD–antibody (RBD-Ab) complex was obtained using:

(3) $$\Delta G_{\mathrm{bind}}=G_{\mathrm{RBD}-\mathrm{AB}}-G_{\mathrm{RBD}}-G_{\mathrm{AB}}$$

(4) $$\Delta G_{bind,MMGBSA}=\Delta E_{MM}+\Delta G_{sol}-T\Delta S$$

where Δ*E_MM_* is total gas phase energy (sum of Δ*E_internal_*, Δ*E_electrostatic_*, and Δ*Evdw*); Δ*G_sol_* is sum of polar (Δ*G_GB_*) and non-polar (Δ*G_SA_*) contributions to solvation. Here, G_RBD–AB_ represents the average over the snapshots of a single trajectory of the complex, G_RBD_ and G_AB_ correspond to the free energy of RBD and Ab protein, respectively.

The polar and non-polar contributions to the solvation-free energy are calculated using a Generalized Born solvent model and consideration of the solvent-accessible surface area [130]. MM-GBSA is employed to predict the binding free energy and decompose the free energy contributions to the binding free energy of a protein–protein complex on a per-residue basis. The binding free energy with MM-GBSA was computed by averaging the results of computations over 1000 samples from the equilibrium ensembles. First, the computational protocol must be selected between the “single-trajectory” (one trajectory of the complex), or “separate-trajectory” (three separate trajectories of the complex, receptor, and ligand). To reduce the noise in the calculations, it is common that each term is evaluated on frames from the trajectory of the bound complex. In this study, we choose the “single-trajectory” protocol because it is less noisy due to the cancellation of intermolecular energy contributions. This protocol applies to cases where significant structural changes upon binding are not expected. Hence, the reorganization energy needed to change the conformational state of the unbound protein and ligand are also not considered. Entropy calculations typically dominate the computational cost of the MM-GBSA estimates. Therefore, it may be calculated only for a subset of the snapshots, or this term can be omitted [131,132]. However, for the absolute affinities, the entropy term is needed, owing to the loss of translational and rotational freedom when the ligand binds. In this study, the entropy contribution was not included in the calculations of binding free energies of the RBD–antibody complexes because the entropic differences in estimates of relative binding affinities are expected to be small [131,132]. MM-GBSA energies were evaluated with the MMPBSA.py script in the AmberTools21 package [133] and gmx_MMPBSA, a tool to perform end-state free energy calculations from CHARMM and GROMACS trajectories [134].

## 4. Conclusions

This study provides a comprehensive and detailed exploration of the molecular interactions between the SARS-CoV-2 S-RBD and neutralizing antibodies from four distinct groups (A–D). By integrating structural analysis, mutational scanning, and MM-GBSA binding free energy calculations, we have uncovered critical insights into the mechanisms of antibody binding, the roles of key residues in stabilizing these interactions, and the evolutionary strategies employed by the virus to evade immune detection. The residue-based decomposition analysis identified critical escape hotspots and provided mechanistic insights into how mutations at these sites reduce antibody binding. Residues such as K417, E484, and K444 are frequently mutated in emerging variants (e.g., K417N, E484K, K444Q) due to their dual roles in binding and immune evasion. These mutations disrupt both van der Waals and electrostatic interactions, leading to significant reductions in binding affinity. Our results dissected the mechanisms underlying group-specific binding and escape profiles. The analysis revealed distinct binding and escape profiles for each antibody group, reflecting their unique epitope targeting and interaction mechanisms. The study highlights the phenomenon of convergent evolution where mutations at key residues (e.g., R346, K444, N460, F486, Q493) emerge independently in multiple variants to modulate antibody binding without compromising ACE2 affinity. The study also highlighted the importance of hinge sites in coordinating the dynamics of antibody binding and viral escape. Hinge positions such as K417, E484, and K444 play a critical role in modulating the conformational dynamics of the RBD. Mutations at these sites can disrupt collective motions and allosteric interactions, reducing the antibody’s ability to bind effectively. The flexibility of hinge regions makes them ideal targets for escape mutations as they allow the virus to rapidly adapt to immune pressure without compromising RBD stability or ACE2 binding.

A comprehensive comparative energetic analysis of group D antibodies suggested that particularly LY-CoV1404 have a strong potential to compete with ACE2 for binding to the RBD due to their engagement with key ACE2-binding residues and their high binding affinity. While AZD1061 and REGN10987 also exhibit competitive binding, their efficacy is more moderate compared to LY-CoV1404. The ability of these antibodies to block ACE2 binding and prevent viral entry makes them valuable tools for therapeutic development. However, the emergence of mutations at critical residues like K444 and V445 highlights the need for continuous adaptation of antibody-based therapies to address evolving viral threats. The findings from this study may have useful implications for the design of next-generation antibodies and therapeutic strategies. Our results suggest that targeting conserved residues like K444 and V445, as seen in group D antibodies, can lead to the development of broadly neutralizing antibodies that are less susceptible to escape mutations. Identifying critical binding hotspots and escape pathways can also guide the rational design of antibodies with improved binding affinity and resilience to viral evolution.

## Data Availability

The original contributions presented in this study are included in the article/Supplementary Materials. Crystal structures were obtained and downloaded from the Protein Data Bank (http://www.rcsb.org (accessed on 20 November 2024)). All simulations were performed using NAMD 2.13 package that was obtained from website https://www.ks.uiuc.edu/Development/Download/ (accessed on 23 November 2024). All simulations were performed using the all-atom additive CHARMM36 protein force field that can be obtained from http://mackerell.umaryland.edu/charmm_ff.shtml (accessed on 25 November 2024). The rendering of protein structures was performed with interactive visualization program UCSF ChimeraX package (https://www.rbvi.ucsf.edu/chimerax/ (accessed on 29 November 2024)) and Pymol (https://pymol.org/2/ (accessed on 29 November 2024)).

## Supplementary Materials

The following supporting information can be downloaded at: https://www.mdpi.com/article/10.3390/ijms26041507/s1.

## Abbreviations

The following abbreviations are used in this manuscript:

MD	Molecular dynamics
NTD	N-terminal domain
RBD	Receptor-binding domain
VOC	Variants of concern
