# Supplementary material for: Mutational Scanning and Binding Free Energy Computations of the SARS-CoV-2 Spike Complexes with Distinct Groups of Neutralizing Antibodies: Energetic Drivers of Convergent Evolution of Binding Affinity and Immune Escape Hotspots
Source: Int J Mol Sci. 2025 Feb 11;26(4):1507. doi: 10.3390/ijms26041507 (PMC11855367; doi:10.3390/ijms26041507)
Supplement: Supplementary file 1 [file ijms-26-01507-s001.zip › ijms-3438317-supplementary.pdf]

Supplementary Materials

Mutational Scanning and Binding Free Energy Computations of the SARS-CoV-2 Spike Complexes with Distinct Groups of Neutralizing Antibodies: Energetic Drivers of Convergent Evolution of Binding Affinity and Immune Escape Hostspots

Mohammed Alshahrani [1], Vedant Parikh [1], Brandon Foley [1], Nishank Raisinghani [1,2] and Gennady Verkhivker [1,3,*]

[1] Keck Center for Science and Engineering, Graduate Program in Computational and Data Sciences, Schmid College of Science and Technology, Chapman University, Orange, CA 92866, USA; alshahrani@chapman.edu (M.A.); vedpar31@gmail.com (V.P.); bfoley@chapman.edu (B.F.); nishankr@stanford.edu (N.R.)

[2] Department of Structural Biology, Stanford University, Stanford, CA 94305, USA

[3] Department of Biomedical and Pharmaceutical Sciences, Chapman University School of Pharmacy, Irvine, CA 92618, USA

* Correspondence: verkhivk@chapman.edu; Tel.: +1-714-516-4586 (G.V)

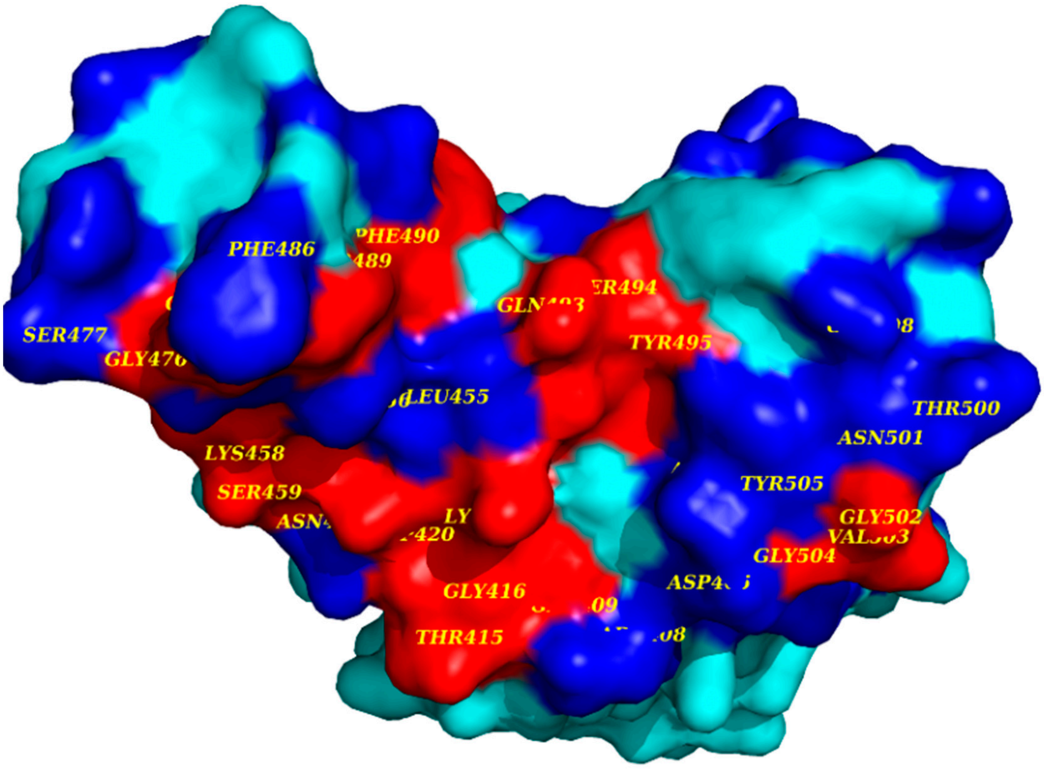

Figure S1. The binding epitope of the group A LY-CoV016-RBD complex. The RBD surface is in cyan, and the binding epitope residues are shown as the red surface. The sites of Omicron lineages are shown as the blue surface (residues 339, 346, 356, 371, 373, 375 376, 403, 405, 408, 417, 440, 444, 445, 446, 450, 452, 455, 456, 460, 475, 477, 478, 481, 484, 486, 493, 498, 501, 505). Binding epitope residues are defined as the RBD binding interface residues that directly interact with antibodies. Residues are considered part of the interface if they are within a defined cutoff distance of 5 Å from atoms in the binding partner.

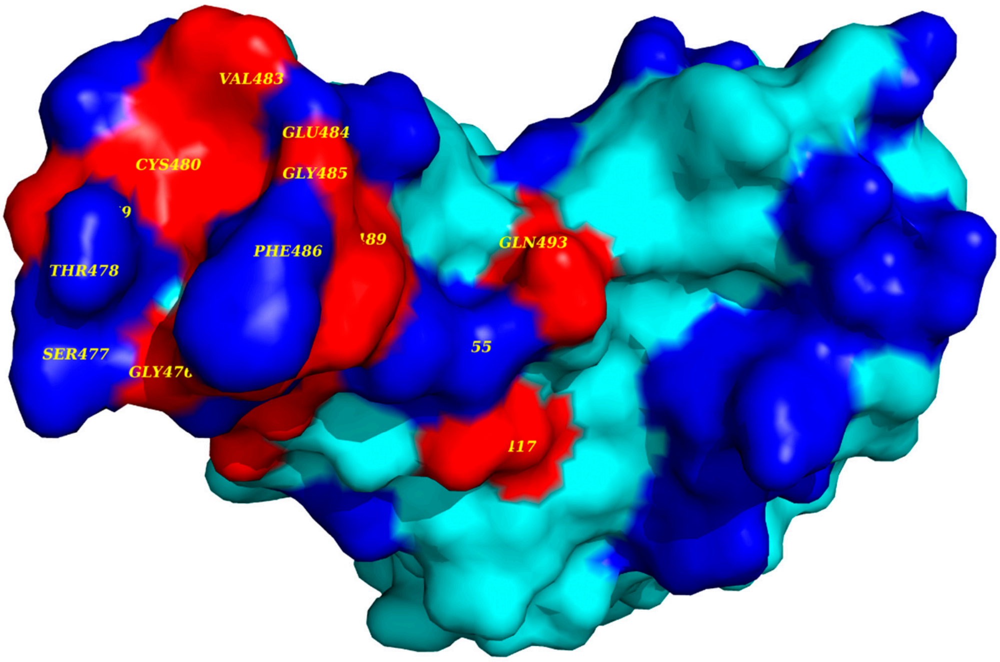

Figure S2. The binding epitope of the group B AZD8895-RBD complex. The RBD surface is in cyan, and the binding epitope residues are shown as the red surface. The sites of Omicron lineages are shown as the blue surface (residues 339, 346, 356, 371, 373, 375 376, 403, 405, 408, 417, 440, 444, 445, 446, 450, 452, 455, 456, 460, 475, 477, 478, 481, 484, 486, 493, 498, 501, 505). Binding epitope residues are defined as the RBD binding interface residues that directly interact with antibodies. Residues are considered part of the interface if they are within a defined cutoff distance of 5 Å from atoms in the binding partner.

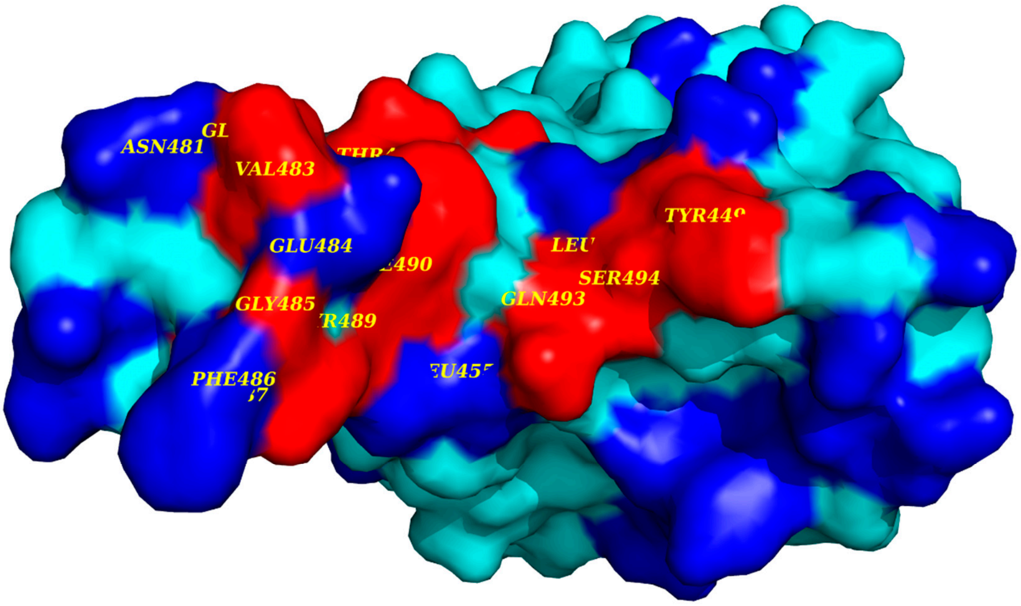

Figure S3. The binding epitope of the group C LY-CoV555-RBD complex. The RBD surface is in cyan, and the binding epitope residues are shown as the red surface. The sites of Omicron lineages are shown as the blue surface (residues 339, 346, 356, 371, 373, 375 376, 403, 405, 408, 417, 440, 444, 445, 446, 450, 452, 455, 456, 460, 475, 477, 478, 481, 484, 486, 493, 498, 501, 505). Binding epitope residues are defined as the RBD binding interface residues that directly interact with antibodies. Residues are considered part of the interface if they are within a defined cutoff distance of 5 Å from atoms in the binding partner.

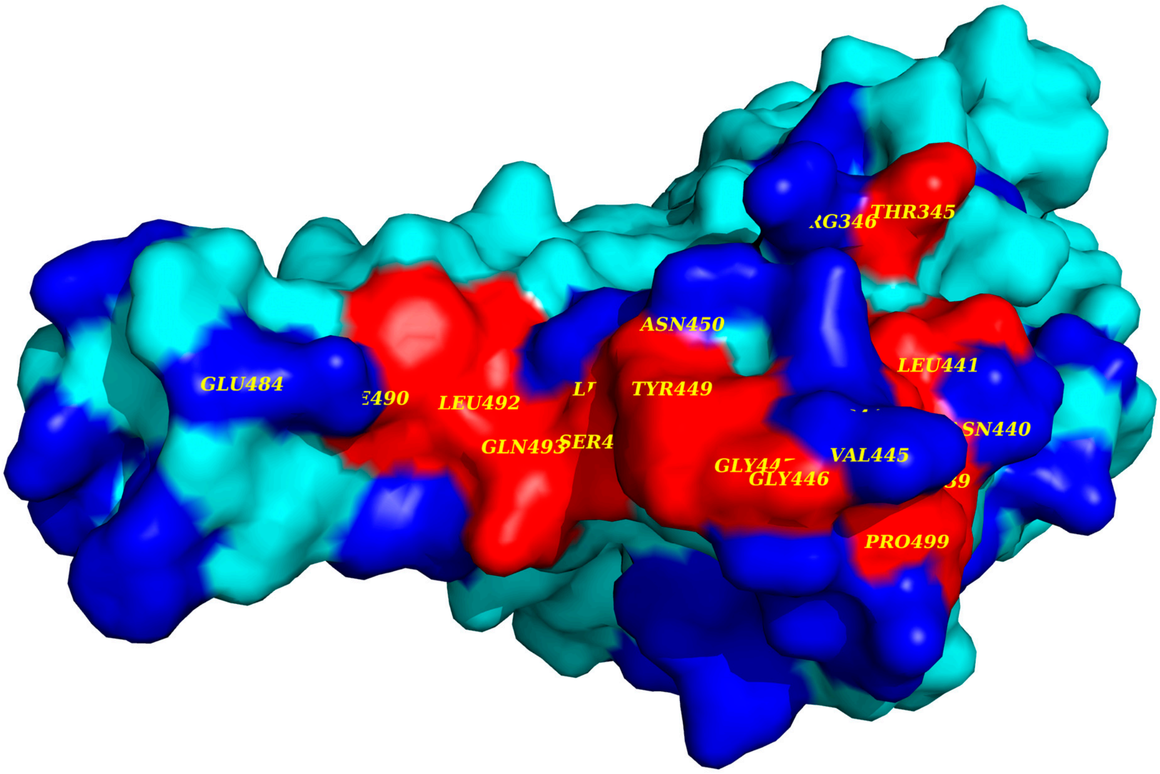

Figure S4. The binding epitope of the group D AZD1061-RBD complex. The RBD surface is in cyan, and the binding epitope residues are shown as the red surface. The sites of Omicron lineages are shown as the blue surface (residues 339, 346, 356, 371, 373, 375 376, 403, 405, 408, 417, 440, 444, 445, 446, 450, 452, 455, 456, 460, 475, 477, 478, 481, 484, 486, 493, 498, 501, 505). Binding epitope residues are defined as the RBD binding interface residues that directly interact with antibodies. Residues are considered part of the interface if they are within a defined cutoff distance of 5 Å from atoms in the binding partner.

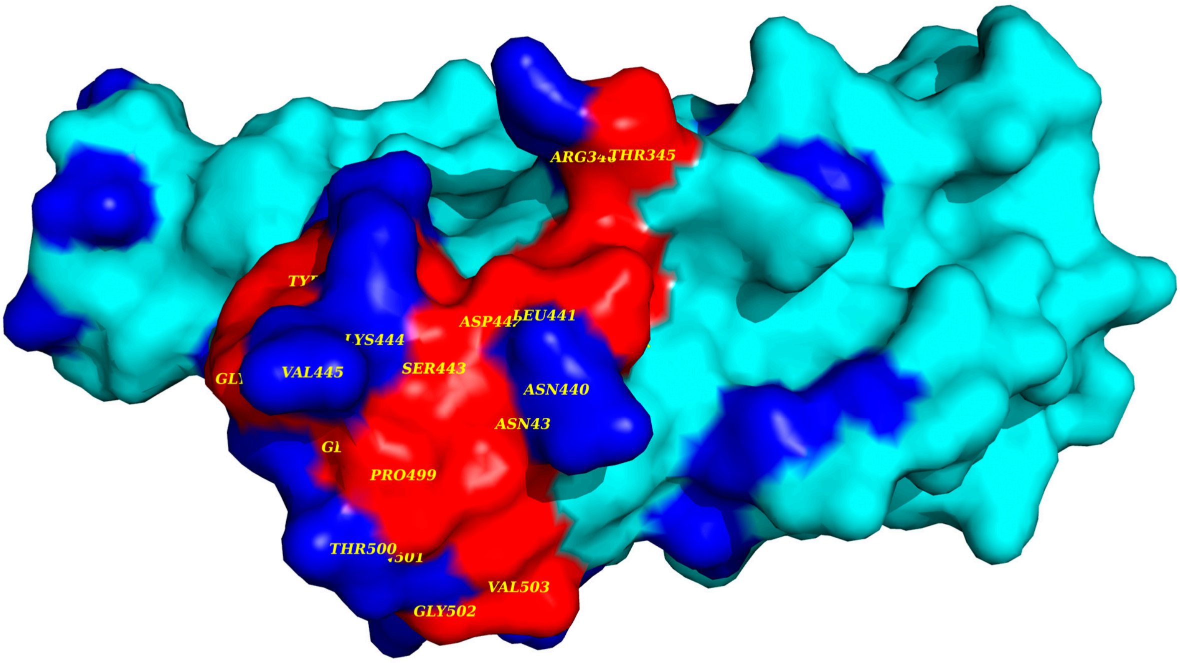

Figure S5. The binding epitope of the group D LY-CoV1404-RBD complex. The RBD surface is in cyan, and the binding epitope residues are shown as the red surface. The sites of Omicron lineages are shown as the blue surface (residues 339, 346, 356, 371, 373, 375 376, 403, 405, 408, 417, 440, 444, 445, 446, 450, 452, 455, 456, 460, 475, 477, 478, 481, 484, 486, 493, 498, 501, 505). Binding epitope residues are defined as the RBD binding interface residues that directly interact with antibodies. Residues are considered part of the interface if they are within a defined cutoff distance of 5 Å from atoms in the binding partner.

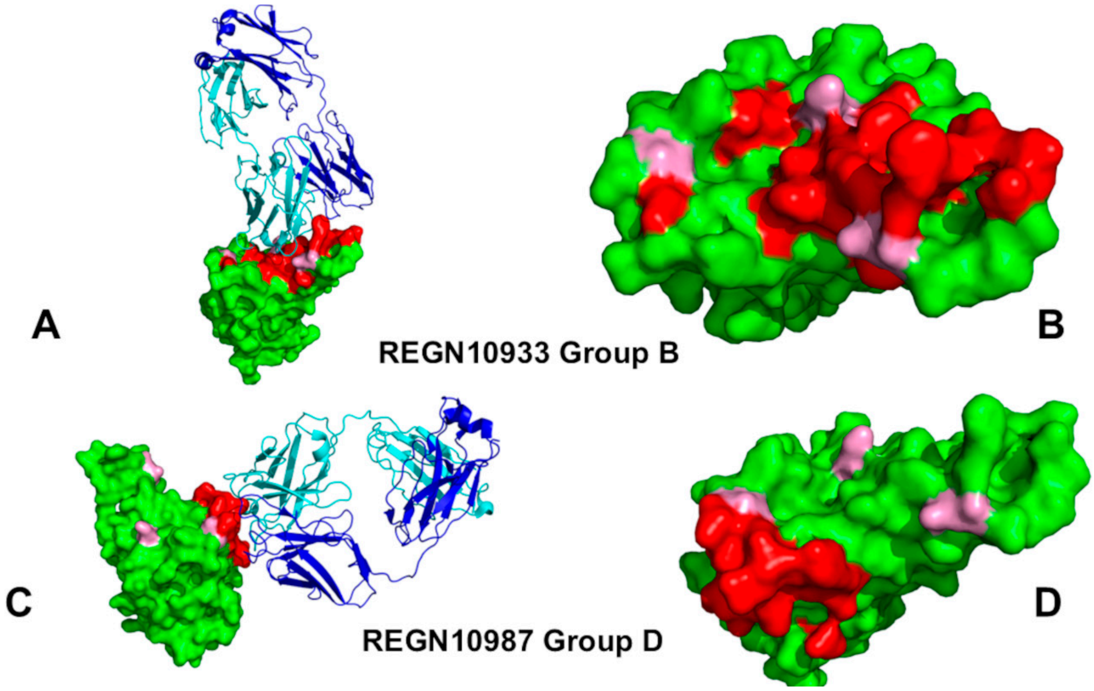

Figure S6. Structure and binding epitopes for the RBD complexes and binding epitopes for group B REGN10933 (A,B) and group D REGN10987 (C,D). The RBD surface is green, and the binding epitope residues are shown as the red surface. The antibody is in ribbons with a heavy chain in magenta and a light chain in light pink. Binding epitope residues are defined as the RBD binding interface residues that directly interact with antibodies. Residues are considered part of the interface if they are within a defined cutoff distance of 5 Å from atoms in the binding partner.

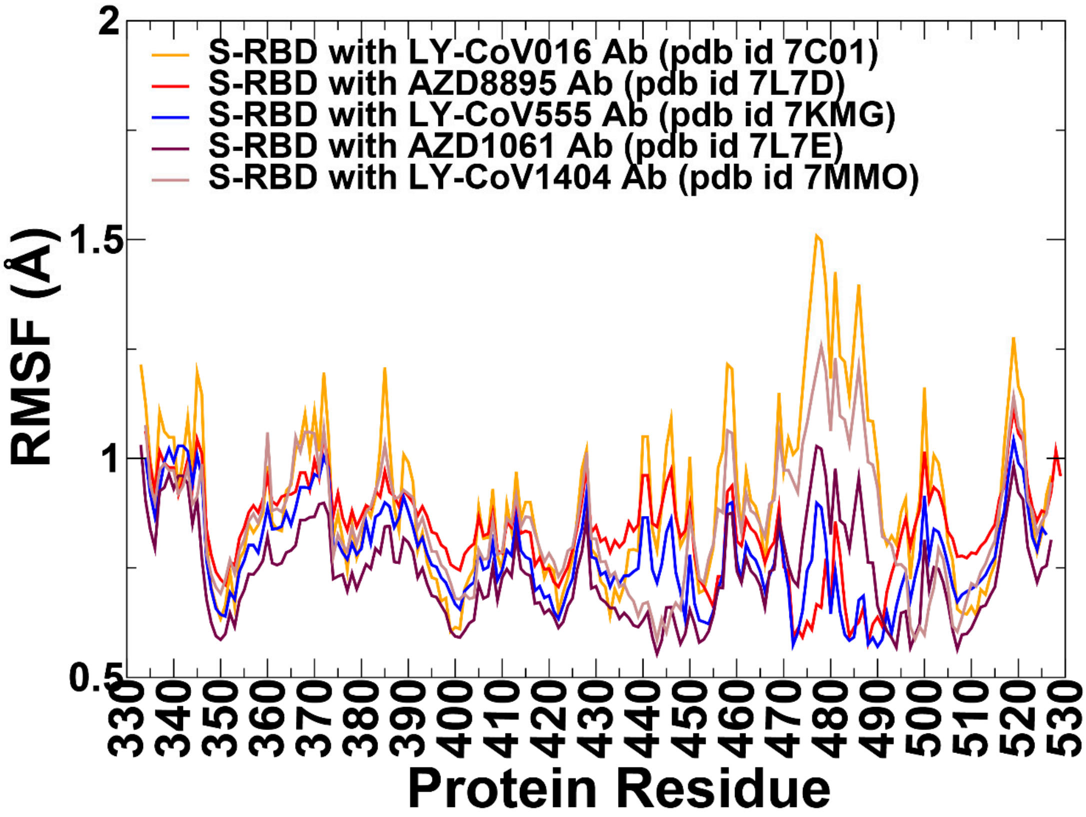

Figure S7. Conformational dynamics profiles obtained from simulations of the RBD–antibody complexes. The RMSF profiles for the RBD residues obtained from MD simulations of the S-RBD complexes with LY-CoV016 (in orange lines), AZD8895 (in red lines), and LY-CoV555 (in blue lines). AZD1061 (in maroon color) and LY-CoV1404 (in brown lines).

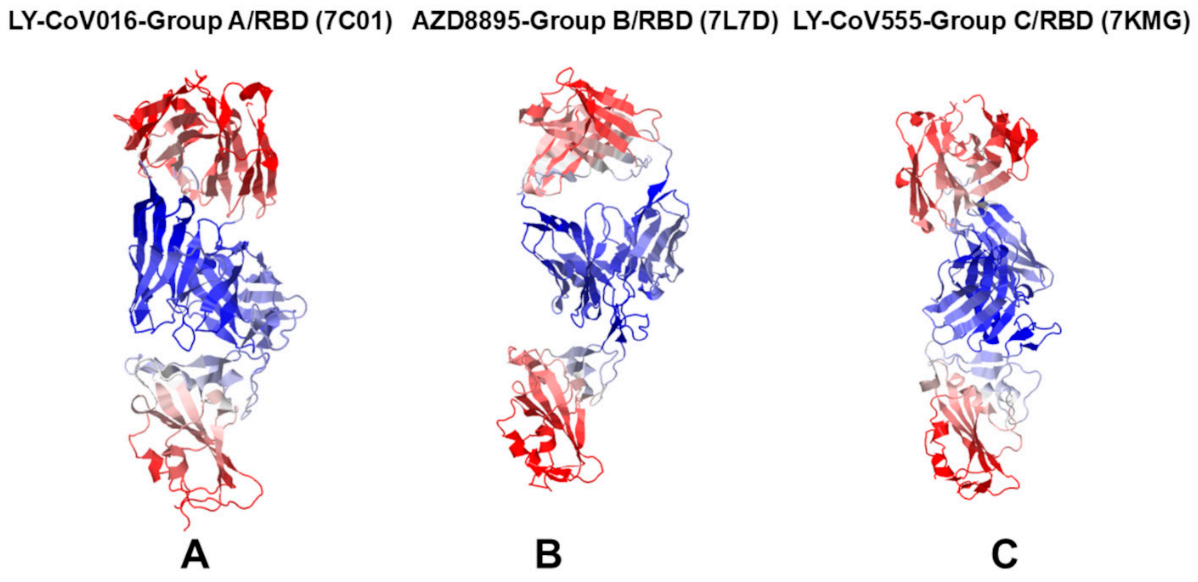

Figure S8. Structural maps of the essential mobility profiles averaged over the slowest three modes for the SARS-CoV-2 S RBD complexes with antibodies. A conformational mobility map for the group A LY-CoV016 complex with RBD (A), group B AZD8895 complex with RBD (B), and group C LY-CoV555 complex with RBD (C). The structures are shown in ribbons with the rigidity–flexibility sliding scale colored from blue (most rigid) to red (most flexible).

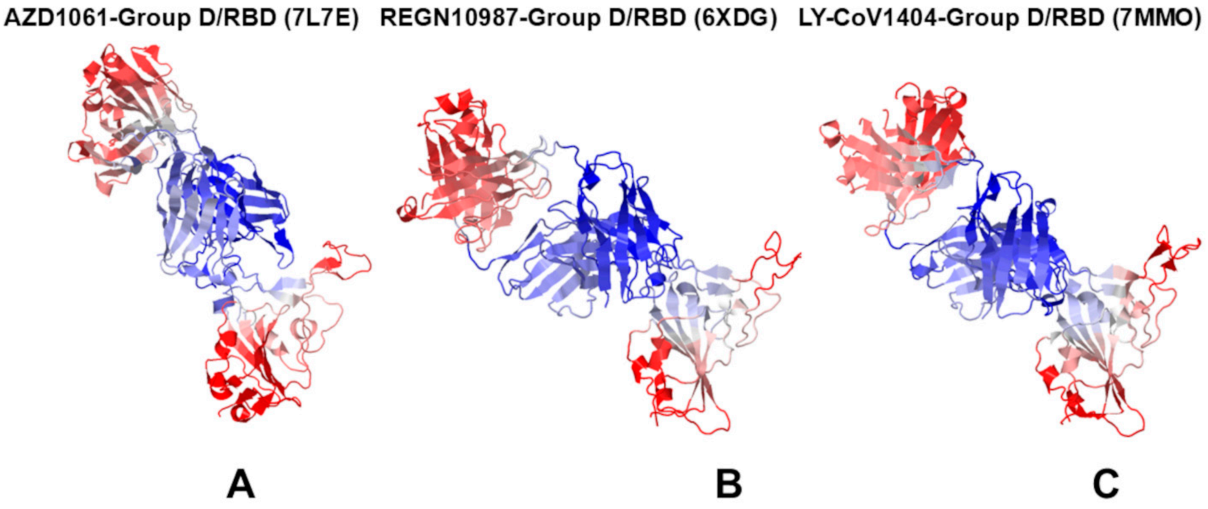

Figure S9. Structural maps of the essential mobility profiles averaged over the slowest three modes for the SARS-CoV-2 S RBD complexes with group D antibodies. A conformational mobility map for the group D AZD1061 complex with RBD (A), group D REGN10987 complex with RBD (B), and group D LY-CoV1404 complex with RBD (C). The structures are shown in ribbons with the rigidity–flexibility sliding scale colored from blue (most rigid) to red (most flexible).

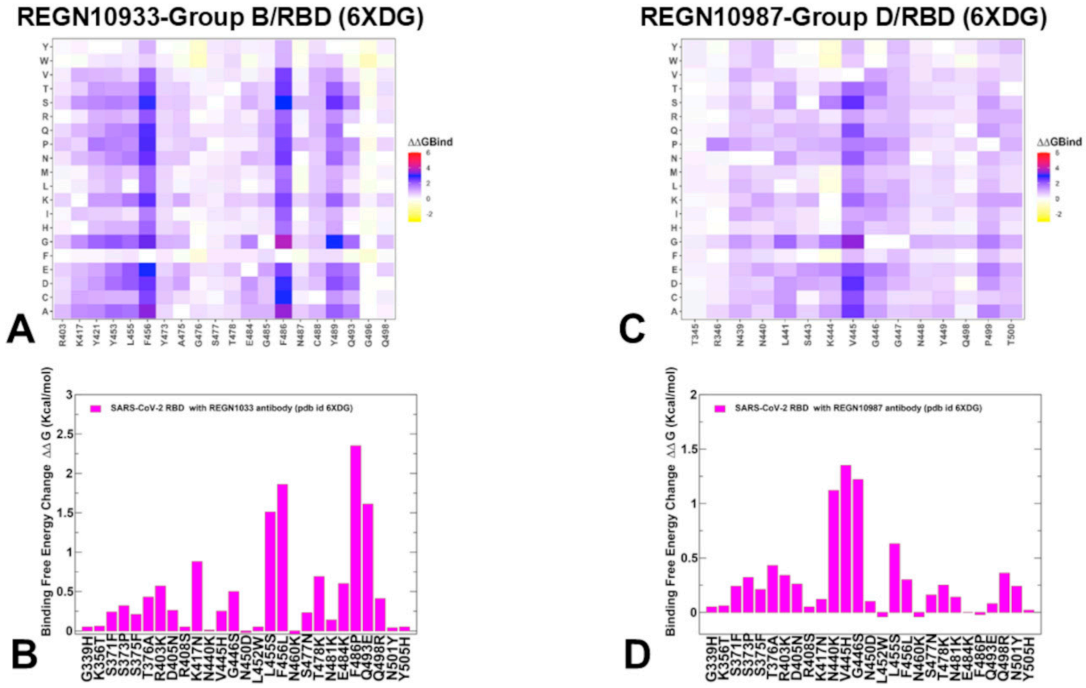

Figure S10. The ensemble-based mutational scanning of binding for the SARS-CoV-2 S-RBD complexes with antibodies REGN10933 (group B) and REGN10987 (group D). The mutational scanning heatmaps for the binding epitope residues in the S-RBD complexes with group B REGN10933 antibody (A) and group D REGN10987 (C). The binding energy hotspots correspond to residues with high mutational sensitivity. The heatmaps show the computed binding free energy changes for 20 single mutations on the sites of variants. The squares on the heatmap are colored using a 3-color scale of blue-white-yellow, with yellow indicating the largest unfavorable effect on stability.

Structure-based mutational profiling distributions of the S-RBD complexes with antibodies REGN10933 (B) and REGN10987 (D). The mutational screening evaluates binding energy changes induced by BA.2.86/JN.1/KP.2/KP.3 mutations in the RBD–antibody complexes. The binding free energy changes are shown in magenta-colored filled bars. The positive binding free energy values ΔΔG correspond to destabilizing changes, and negative binding free energy changes are associated with stabilizing changes.

Table S1. The list of the binding epitope residues for group A LY-CoV016 antibody complex with RBD

RBD residue	RBD residue number	RBD chain	Antibody residue	Antibody residue number	Antibody chain
ARG	403	A	TYR	32	L
ARG	403	A	THR	94	L
ARG	403	A	SER	93	L
ARG	403	A	TYR	92	L
ASP	405	A	THR	94	L
ASP	405	A	SER	93	L
ASP	405	A	PRO	95	L
ASP	405	A	TYR	92	L
GLU	406	A	THR	94	L
ARG	408	A	PRO	95	L
ARG	408	A	THR	94	L
ARG	408	A	PHE	58	H
GLN	409	A	THR	94	L
THR	415	A	PHE	58	H
THR	415	A	SER	56	H
THR	415	A	THR	57	H
GLY	416	A	PHE	58	H
GLY	416	A	TYR	52	H
GLY	416	A	SER	56	H
LYS	417	A	ASP	104	H
LYS	417	A	TYR	33	H
LYS	417	A	TYR	52	H
LYS	417	A	PRO	100	H
LYS	417	A	GLY	103	H
ASP	420	A	SER	56	H
ASP	420	A	PHE	58	H
ASP	420	A	TYR	52	H
TYR	421	A	TYR	33	H
TYR	421	A	GLY	54	H
TYR	421	A	GLY	55	H
TYR	421	A	SER	53	H
TYR	421	A	TYR	52	H
TYR	453	A	TYR	32	L
TYR	453	A	TYR	102	H
TYR	453	A	PRO	100	H
TYR	453	A	GLY	103	H
LEU	455	A	SER	53	H
LEU	455	A	PRO	100	H

LEU	455	A	TYR	33	H
LEU	455	A	MET	101	H
LEU	455	A	TYR	102	H
PHE	456	A	MET	101	H
PHE	456	A	VAL	98	H
PHE	456	A	ASN	32	H
PHE	456	A	SER	31	H
PHE	456	A	SER	53	H
PHE	456	A	PRO	100	H
PHE	456	A	LEU	99	H
PHE	456	A	TYR	33	H
ARG	457	A	GLY	54	H
ARG	457	A	SER	53	H
LYS	458	A	GLY	54	H
LYS	458	A	SER	31	H
LYS	458	A	SER	53	H
LYS	458	A	ARG	71	H
LYS	458	A	SER	30	H
SER	459	A	SER	53	H
SER	459	A	GLY	54	H
ASN	460	A	SER	56	H
ASN	460	A	GLY	54	H
ASN	460	A	GLY	55	H
TYR	473	A	SER	31	H
TYR	473	A	SER	53	H
TYR	473	A	ASN	32	H
TYR	473	A	SER	30	H
GLN	474	A	SER	31	H
ALA	475	A	PHE	27	H
ALA	475	A	ARG	97	H
ALA	475	A	ASN	32	H
ALA	475	A	THR	28	H
ALA	475	A	SER	31	H
GLY	476	A	ASN	32	H
GLY	476	A	THR	28	H
GLY	476	A	SER	31	H
GLY	476	A	PHE	27	H
GLY	476	A	GLY	26	H
SER	477	A	PHE	27	H
SER	477	A	THR	28	H
PHE	486	A	ASP	107	H
PHE	486	A	VAL	2	H
PHE	486	A	GLU	1	H

PHE	486	A	GLY	26	H
PHE	486	A	TYR	108	H
PHE	486	A	ARG	97	H
ASN	487	A	ARG	97	H
ASN	487	A	ASN	32	H
ASN	487	A	PHE	27	H
ASN	487	A	GLY	26	H
TYR	489	A	ARG	97	H
TYR	489	A	ASP	107	H
TYR	489	A	LEU	99	H
TYR	489	A	MET	101	H
TYR	489	A	ASN	32	H
PHE	490	A	MET	101	H
GLN	493	A	TYR	102	H
GLN	493	A	PRO	100	H
GLN	493	A	GLY	103	H
GLN	493	A	MET	101	H
SER	494	A	TYR	32	L
TYR	495	A	TYR	32	L
GLN	498	A	SER	30	L
THR	500	A	SER	28	L
ASN	501	A	SER	30	L
ASN	501	A	SER	28	L
ASN	501	A	TYR	92	L
GLY	502	A	SER	28	L
GLY	502	A	TYR	92	L
VAL	503	A	TYR	92	L
GLY	504	A	TYR	92	L
TYR	505	A	SER	30	L
TYR	505	A	TYR	92	L
TYR	505	A	TYR	32	L

Table S2. The list of the binding epitope residues for group B AZD8895 antibody complex with RBD

RBD residue	RBD residue number	RBD chain	Antibody residue	Antibody residue number	Antibody chain
LYS	417	E	GLY	54	H
LEU	455	E	SER	55	H
LEU	455	E	GLY	54	H
PHE	456	E	MET	30	H
PHE	456	E	GLY	54	H
PHE	456	E	VAL	52	H
PHE	456	E	SER	55	H
LYS	458	E	ILE	104	H
TYR	473	E	ILE	104	H
ALA	475	E	ILE	104	H
ALA	475	E	SER	31	H
ALA	475	E	CYS	106	H
ALA	475	E	SER	105	H
GLY	476	E	ASN	107	H
GLY	476	E	SER	105	H
GLY	476	E	CYS	106	H
GLY	476	E	ASP	108	H
SER	477	E	SER	105	H
SER	477	E	CYS	106	H
SER	477	E	ASP	108	H
THR	478	E	TYR	33	L
THR	478	E	SER	32	L
THR	478	E	ASP	108	H
PRO	479	E	TYR	33	L
CYS	480	E	TYR	33	L
VAL	483	E	SER	94	L
GLU	484	E	SER	95	L
GLU	484	E	SER	94	L
GLY	485	E	SER	95	L
GLY	485	E	TRP	98	L
GLY	485	E	SER	94	L
GLY	485	E	TRP	50	H
PHE	486	E	PRO	99	H
PHE	486	E	TYR	92	L
PHE	486	E	ASP	108	H
PHE	486	E	TRP	50	H
PHE	486	E	ASN	107	H
PHE	486	E	TRP	98	L
PHE	486	E	SER	95	L

PHE	486	E	SER	94	L
PHE	486	E	ALA	33	H
PHE	486	E	GLY	109	H
PHE	486	E	TYR	33	L
PHE	486	E	PHE	110	H
ASN	487	E	CYS	101	H
ASN	487	E	PRO	99	H
ASN	487	E	CYS	106	H
ASN	487	E	ASP	108	H
ASN	487	E	TRP	50	H
ASN	487	E	SER	31	H
ASN	487	E	ASN	107	H
ASN	487	E	GLY	109	H
CYS	488	E	SER	95	L
CYS	488	E	TYR	33	L
TYR	489	E	TRP	50	H
TYR	489	E	ALA	33	H
TYR	489	E	SER	31	H
TYR	489	E	VAL	52	H
TYR	489	E	SER	32	H
GLN	493	E	ASN	57	H
GLN	493	E	SER	55	H
GLN	493	E	GLY	56	H
GLN	493	E	GLY	54	H

Table S3. The list of the binding epitope residues for group B REGN10933 antibody complex with RBD

RBD residue	RBD residue number	RBD chain	Antibody residue	Antibody residue number	Antibody chain
ARG	403	E	THR	28	B
GLU	406	E	THR	28	B
LYS	417	E	ASP	31	B
LYS	417	E	THR	28	B
LYS	417	E	TYR	32	B
LYS	417	E	THR	102	B
TYR	421	E	THR	103	B
TYR	421	E	THR	102	B
TYR	453	E	SER	30	B
TYR	453	E	ASP	31	B
LEU	455	E	TYR	32	B
LEU	455	E	SER	30	B
LEU	455	E	ASP	31	B
LEU	455	E	THR	102	B
PHE	456	E	THR	103	B
PHE	456	E	GLY	101	B
PHE	456	E	ASP	31	B
PHE	456	E	MET	104	B
PHE	456	E	THR	102	B
PHE	456	E	ARG	100	B
TYR	473	E	MET	104	B
ALA	475	E	TYR	32	D
ALA	475	E	MET	104	B
GLY	476	E	TYR	32	D
GLY	476	E	ASP	92	D
SER	477	E	TYR	32	D
SER	477	E	ASP	92	D
THR	478	E	ASN	93	D
GLU	484	E	THR	57	B
GLU	484	E	SER	54	B
GLU	484	E	SER	56	B
GLU	484	E	THR	52	B
GLU	484	E	TYR	59	B
GLU	484	E	TYR	53	B
GLY	485	E	TYR	33	B
GLY	485	E	THR	52	B
GLY	485	E	TYR	59	B
GLY	485	E	THR	57	B

PHE	486	E	TYR	91	D
PHE	486	E	THR	57	B
PHE	486	E	LEU	94	D
PHE	486	E	LEU	96	D
PHE	486	E	ASP	92	D
PHE	486	E	TYR	33	B
PHE	486	E	ASN	93	D
PHE	486	E	TYR	50	B
PHE	486	E	TYR	59	B
PHE	486	E	ARG	100	B
ASN	487	E	ASP	92	D
ASN	487	E	TYR	59	B
ASN	487	E	ARG	100	B
ASN	487	E	TYR	33	B
CYS	488	E	ARG	100	B
CYS	488	E	TYR	33	B
TYR	489	E	TYR	32	B
TYR	489	E	TYR	33	B
TYR	489	E	SER	30	B
TYR	489	E	THR	52	B
TYR	489	E	ASP	31	B
TYR	489	E	TYR	53	B
PHE	490	E	TYR	53	B
LEU	492	E	TYR	53	B
GLN	493	E	ASN	74	B
GLN	493	E	TYR	53	B
GLN	493	E	SER	54	B
GLN	493	E	SER	30	B
GLN	493	E	ASP	31	B
SER	494	E	ASN	74	B
GLN	498	E	ALA	75	B

Table S4. The list of the binding epitope residues for group C LY-CoV555 antibody complex with RBD

RBD residue	RBD residue number	RBD chain	Antibody residue	Antibody residue number	Antibody chain
TYR	351	C	LEU	55	A
TYR	449	C	ASN	31	A
TYR	449	C	ILE	54	A
TYR	449	C	SER	30	A
TYR	449	C	GLU	102	A
LEU	452	C	ILE	54	A
LEU	452	C	GLU	102	A
LEU	452	C	LEU	55	A
LEU	455	C	ARG	104	A
LEU	455	C	ALA	103	A
PHE	456	C	ALA	103	A
THR	470	C	LEU	55	A
THR	470	C	ILE	57	A
ILE	472	C	ARG	50	A
ASN	481	C	THR	94	B
ASN	481	C	ASN	59	A
GLY	482	C	ASN	59	A
GLY	482	C	ARG	50	A
VAL	483	C	ASN	59	A
VAL	483	C	TYR	60	A
VAL	483	C	ARG	50	A
VAL	483	C	ARG	96	B
VAL	483	C	TRP	47	A
VAL	483	C	THR	94	B
GLU	484	C	TYR	92	B
GLU	484	C	ARG	50	A
GLU	484	C	TYR	101	A
GLU	484	C	TYR	110	A
GLU	484	C	SER	91	B
GLU	484	C	ALA	33	A
GLU	484	C	ILE	52	A
GLU	484	C	ARG	96	B
GLU	484	C	TYR	100	A
GLY	485	C	ARG	96	B
GLY	485	C	TYR	32	B
GLY	485	C	TYR	92	B
GLY	485	C	SER	93	B
GLY	485	C	TYR	110	A
GLY	485	C	SER	91	B

PHE	486	C	SER	30	B
PHE	486	C	SER	91	B
PHE	486	C	TYR	32	B
PHE	486	C	TYR	92	B
PHE	486	C	SER	93	B
PHE	486	C	TYR	110	A
ASN	487	C	TYR	32	B
CYS	488	C	TYR	110	A
TYR	489	C	TYR	32	B
TYR	489	C	TYR	110	A
PHE	490	C	ALA	103	A
PHE	490	C	TYR	110	A
PHE	490	C	GLU	102	A
PHE	490	C	ILE	52	A
PHE	490	C	ILE	57	A
PHE	490	C	LEU	55	A
PHE	490	C	ARG	50	A
PHE	490	C	TYR	101	A
LEU	492	C	TYR	101	A
LEU	492	C	GLU	102	A
LEU	492	C	LEU	55	A
GLN	493	C	ALA	103	A
GLN	493	C	GLU	102	A
GLN	493	C	HIS	105	A
GLN	493	C	ARG	104	A
SER	494	C	ASN	31	A
SER	494	C	GLU	102	A

Table S5. The list of the binding epitope residues for group D AZD1061 complex with RBD

RBD residue	RBD residue number	RBD chain	Antibody residue	Antibody residue number	Antibody chain
THR	345	G	THR	108	E
THR	345	G	ILE	55	E
ARG	346	G	ILE	55	E
ARG	346	G	TYR	106	E
ARG	346	G	ASP	56	E
ARG	346	G	THR	108	E
ARG	346	G	ASP	107	E
ARG	346	G	TRP	33	E
ASN	439	G	PRO	111	E
ASN	440	G	GLY	112	E
ASN	440	G	PRO	111	E
ASN	440	G	GLY	110	E
LEU	441	G	GLY	110	E
LEU	441	G	PRO	111	E
LEU	441	G	THR	108	E
LEU	441	G	VAL	109	E
ASP	442	G	PRO	111	E
ASP	442	G	GLY	110	E
SER	443	G	TYR	104	E
SER	443	G	GLY	110	E
SER	443	G	PRO	111	E
SER	443	G	VAL	109	E
LYS	444	G	GLY	110	E
LYS	444	G	PRO	111	E
LYS	444	G	THR	108	E
LYS	444	G	VAL	109	E
LYS	444	G	ASP	107	E
LYS	444	G	TYR	104	E
LYS	444	G	SER	103	E
LYS	444	G	TYR	106	E
LYS	444	G	TYR	105	E
VAL	445	G	GLY	116	E
VAL	445	G	LEU	113	E
VAL	445	G	PRO	111	E
VAL	445	G	TRP	56	F
VAL	445	G	GLY	110	E

VAL	445	G	TYR	55	F
VAL	445	G	TYR	104	E
VAL	445	G	PHE	118	E
VAL	445	G	GLU	61	F
GLY	446	G	THR	59	F
GLY	446	G	TRP	56	F
GLY	446	G	TYR	55	F
GLY	446	G	LYS	36	F
GLY	447	G	TRP	56	F
TYR	449	G	ASN	34	F
TYR	449	G	TYR	38	F
TYR	449	G	TRP	56	F
TYR	449	G	LYS	36	F
ASN	450	G	TYR	38	F
ASN	450	G	ASP	107	E
ASN	450	G	TYR	106	E
ASN	450	G	ASN	34	F
ASN	450	G	TYR	105	E
LEU	452	G	SER	33	F
LEU	452	G	ASN	34	F
GLU	484	G	SER	32	F
PHE	490	G	SER	33	F
PHE	490	G	SER	32	F
LEU	492	G	SER	33	F
LEU	492	G	SER	32	F
GLN	493	G	SER	33	F
GLN	493	G	ASN	34	F
SER	494	G	SER	33	F
SER	494	G	ASN	34	F
PRO	499	G	PRO	111	E

Table S6. The list of the binding epitope residues for group D REGN10987 complex with RBD

RBD residue	RBD residue number	RBD chain	Antibody residue	Antibody residue number	Antibody chain
ARG	346	E	ASN	31	C
ASN	439	E	GLY	103	C
ASN	439	E	ASP	104	C
ASN	440	E	ASP	101	C
ASN	440	E	GLY	103	C
ASN	440	E	TYR	102	C
ASN	440	E	ASP	104	C
LEU	441	E	ASP	101	C
LEU	441	E	GLY	103	C
LEU	441	E	TYR	102	C
SER	443	E	ASP	104	C
SER	443	E	GLY	103	C
LYS	444	E	TYR	32	C
LYS	444	E	ASN	31	C
LYS	444	E	TYR	53	C
VAL	445	E	SER	52	C
VAL	445	E	ASN	57	C
VAL	445	E	TRP	99	A
VAL	445	E	TYR	53	C
VAL	445	E	ILE	51	C
VAL	445	E	TYR	59	C
VAL	445	E	TYR	105	C
VAL	445	E	TYR	35	C
VAL	445	E	VAL	50	C
VAL	445	E	ALA	33	C
GLY	446	E	TYR	59	C
GLY	446	E	SER	52	C
GLY	446	E	ASN	57	C
GLY	447	E	TYR	53	C
GLY	447	E	TYR	59	C
GLY	447	E	ASP	54	C
GLY	447	E	SER	52	C
GLY	447	E	ASN	57	C
ASN	448	E	TYR	53	C
TYR	449	E	TYR	53	C
TYR	449	E	SER	56	C
TYR	449	E	ASP	54	C
TYR	449	E	ASN	57	C

ASN	450	E	TYR	53	C
GLN	498	E	TYR	59	C
PRO	499	E	LEU	93	A
PRO	499	E	TYR	34	A
PRO	499	E	ASP	104	C
PRO	499	E	TYR	105	C
THR	500	E	TYR	32	A
THR	500	E	TRP	99	A
THR	500	E	LEU	93	A
THR	500	E	SER	95	A
ASN	501	E	TYR	32	A

Table S7. The list of the binding epitope residues for group D LY-CoV1404 complex with RBD

RBD residue	RBD residue number	RBD chain	Antibody residue	Antibody residue number	Antibody chain
THR	345	C	SER	32	A
THR	345	C	ILE	31	A
ARG	346	C	SER	30	A
ARG	346	C	TRP	55	A
ARG	346	C	ILE	31	A
ARG	346	C	SER	32	A
ASN	439	C	TYR	35	B
ASN	439	C	ILE	102	A
ASN	440	C	TYR	35	B
ASN	440	C	GLY	33	A
ASN	440	C	SER	103	A
ASN	440	C	ILE	102	A
ASN	440	C	GLU	53	B
LEU	441	C	TRP	55	A
LEU	441	C	SER	32	A
LEU	441	C	GLY	33	A
LEU	441	C	ILE	102	A
ASP	442	C	SER	32	A
SER	443	C	ILE	102	A
SER	443	C	TYR	54	A
LYS	444	C	ASP	58	A
LYS	444	C	TRP	55	A
LYS	444	C	ASP	56	A
LYS	444	C	ARG	60	A
LYS	444	C	ILE	102	A
LYS	444	C	TYR	54	A
VAL	445	C	HIS	100	A
VAL	445	C	TRP	49	A
VAL	445	C	SER	98	B
VAL	445	C	ALA	99	B
VAL	445	C	THR	96	B
VAL	445	C	ARG	60	A
VAL	445	C	SER	97	B
VAL	445	C	ILE	102	A
VAL	445	C	TYR	54	A
VAL	445	C	TYR	94	B
VAL	445	C	LEU	52	A
GLY	446	C	ARG	60	A
GLY	446	C	SER	97	B

GLY	446	C	THR	96	B
GLY	446	C	SER	98	B
GLY	446	C	THR	95	B
GLY	447	C	ASP	58	A
GLY	447	C	THR	96	B
GLY	447	C	ARG	60	A
ASN	448	C	TRP	55	A
ASN	448	C	ASP	56	A
ASN	448	C	ARG	60	A
ASN	448	C	ASP	58	A
TYR	449	C	ASP	58	A
TYR	449	C	ARG	60	A
ASN	450	C	ASP	56	A
ASN	450	C	ASP	58	A
ASN	450	C	TRP	55	A
GLN	498	C	THR	95	B
GLN	498	C	THR	96	B
GLN	498	C	SER	97	B
PRO	499	C	THR	95	B
PRO	499	C	TYR	35	B
PRO	499	C	ILE	102	A
PRO	499	C	TYR	94	B
PRO	499	C	ASN	34	B
THR	500	C	TYR	94	B
THR	500	C	TYR	33	B
THR	500	C	GLY	31	B
THR	500	C	THR	95	B
THR	500	C	ASP	32	B
THR	500	C	THR	96	B
THR	500	C	ASP	29	B
THR	500	C	VAL	30	B
ASN	501	C	ASP	32	B
ASN	501	C	GLY	31	B
ASN	501	C	ASN	34	B
GLY	502	C	ASP	32	B
GLY	502	C	ASN	34	B
VAL	503	C	ASN	34	B
VAL	503	C	ASP	32	B
GLN	506	C	ASN	34	B
GLN	506	C	TYR	35	B
GLN	506	C	ASP	32	B
ARG	509	C	SER	32	A
